# Macrophage miR-4524a-5p/TBP promotes β-TrCP -TIM3 complex activation and TGFβ release and aggravates NAFLD-associated fibrosis

**DOI:** 10.1038/s41419-025-07574-4

**Published:** 2025-04-19

**Authors:** Chunming Li, Lei Fang, Xingxing Su, Jie Zhang, Haojun Xiong, Hongqiang Yu, Zhu Zhu, Xiaotong Lin, Ke Min, Di Wu, Zhiyu Chen, Jianping Gong, Chuan-Ming Xie

**Affiliations:** 1 Key Laboratory of Hepatobiliary and Pancreatic Surgery, Institute of Hepatobiliary Surgery, Southwest Hospital, Third Military Medical University (Army Medical University), Chongqing, China; 2 Department of Hepatobiliary Surgery, The Second Affiliated Hospital of Chongqing Medical University, Chongqing, China

**Keywords:** Non-alcoholic fatty liver disease, Predictive markers, Prognostic markers, Translational research

## Abstract

Macrophages hold a critical position in maintenance of hepatic homeostasis and in injury and repair processes in acute and chronic liver diseases. TIM3 is a promising protector in MCD-induced steatohepatitis in acute liver injury. However, we recently find TIM3 as a driver of fibrosis in MCD/HFD-induced chronic liver injury. This study aims to explore how macrophage TIM3 drivers NAFLD-associated chronic liver injury as well as identify a subtype of fibrotic patients suitable for anti-TIM3 immunotherapy. Here, we found that TIM3 was highly expressed in liver macrophages in a long-term MCD- or HFD-fed mice with fibrotic NASH. Elevated β-TrCP in macrophages promoted TIM3 polyubiquitination and membrane translocation. The ubiquitinated TIM3 then bound with PI3K and followed by inhibition of mTOR and activation of macrophage M2 polarization and TGF-β release, leading to HSC activation and liver fibrosis. Furthermore, elevated TIM3 was attributed to the transcriptional TBP upregulation and miR-4524a-5p downregulation. Targeting of TIM3 significantly attenuated liver fibrosis in mice. In clinical NASH patients, elevated macrophage TIM3 is positively correlated with TBP expression and negatively associated with miR-4524a-5p. Decreased miR-4524a-5p in plasma was a biomarker for the NASH fibrosis patients suitable for anti-TIM3 therapy. In conclusion, this study reveals that miR-4524a-5p/TBP promotes β-TrCP/TIM3 complex activation in macrophages and aggravates chronic NASH fibrosis, providing miR-4524a-5p as an effective blood biomarker for a subtype of chronic NASH patients with fibrosis suitable for anti-TIM3 treatment.

## Introduction

Nonalcoholic fatty liver disease (NAFLD), also recently referred to as metabolic dysfunction-associated fatty liver disease (MAFLD), is a chronic liver disease characterized by the accumulation of triglycerides (TAGs) in hepatocytes and affects approximately 25-30% of adults worldwide [1, 2]. Fibrotic deposits reflect a relatively more stable histologic feature and are the strongest predictor of liver-related and all-cause mortality independent of any other histopathological features [3, 4]. Preventing the occurrence and progression of fibrosis is the key to preventing and treating NAFLD-related cirrhosis and HCC. However, due to our incomplete understanding of the mechanisms of fibrotic NASH progression, there are no FDA-approved drugs to treat NASH.

The immune microenvironment in fibrotic NASH progression remains unclear. T-cell immunoglobulin mucin-3 (TIM3), a novel immune checkpoint molecule encoded by the Havcr2 gene, has attracted increasing attention as a potential immunotherapeutic target for different diseases. TIM3 was first found to be expressed on the surface of Th1 cells and later on the surface of other subtypes of T cells, monocytes, and macrophages [5–7]. In a mouse HCC model, TIM3 overexpression on tumor-associated macrophages (TAMs) inhibited the activation of tumor-specific CD8 + T cells [8]. TIM3 was found to negatively regulate early steatohepatitis in MCD-fed 2-week-old mice [9]. We currently found that TIM3 functions as a driver of fibrosis and promotes MCD/HFD-fed chronic liver fibrosis. However, how TIM3 is mechanistically linked to NASH fibrosis remains unknown.

In this study, we found that TIM3 in macrophages was increased in chronic NASH with fibrosis. β-TrCP promoted the elevated TIM3 polyubiquitination and followed by relocalization onto membrane. Elevated TIM3 induced the conversion of macrophages to a profibrotic phenotype and increased the secretion of TGF-β by inhibiting the mTOR signaling pathway, thereby promoting the proliferation and activation of HSCs and fibrosis. The decreased miR-4524a-5p promoted transcription factor TBP accumulation, followed by TIM3 upregulation in macrophages. Targeting of TIM3 suppressed NASH fibrosis in mice. These findings provide a potential mechanism through which TIM3 is highly expressed and activated in macrophages and causes HSC activation and NASH fibrosis, highlighting a possible therapeutic strategy for the treatment of fibrosis in chronic NASH patients.

## Methods

### Patients and tissue samples

Forty-eight patients with biopsy-proven nonalcoholic fatty liver disease admitted to the Department of Hepatobiliary Surgery, the First Affiliated Hospital of Army Military Medical University, were enrolled in this study. The specific clinicopathological characteristics of NAFLD patients are shown in Supplementary Table 1. After obtaining the consent of the patients and their families, we signed the informed consent form for clinical sample collection. This study was approved by the Ethics Committee of the First Affiliated Hospital of Army Military Medical University (KY2022124; KY2020127) and was in accordance with the Declaration of Helsinki.

### Animal study

Wild-type (WT) C57BL/6J mice were purchased from GemPharmatech Co., Ltd. (Chengdu, China). Havcr2^fl/+^; Lyz2-cre and TIM3 knockdown (C57BL/6J background, Havcr2^+/−^) mice were generated by Cyagen Biosciences (Suzhou, China). Eight- to ten-week-old male mice were fed a methionine and choline-deficient (MCD) diet (MD12052, Medison, Jiangsu, China) for 2 weeks for the NAFLD model and 5 weeks for the NASH fibrosis model. Four-week-old male WT mice were fed a high-fat diet (HFD) (MD12032, Medison, Jiangsu, China) for 26 weeks to build a NASH fibrosis model. For TIM3 treatment, all animals were simultaneously randomized to the treatment groups without considering any other variable, and anti-TIM-3 IgG (RMT3-23, BioXCell) was administered by intraperitoneal injection at a dose of 0.5 mg/mouse every 5 days for 3 weeks. We selected a small sample size because targeting of TIM3 in NASH fibrosis was evaluated in vivo for the first time in the present study. For each animal, three different investigators were involved as follows: a first investigator administered the treatment based on the randomization group. A second investigator was responsible for the sample harvesting and outcome measure, whereas a third investigator performed the data analysis. All mice were treated in the Experimental Animal Center of the First Affiliated Hospital of Army Medical University (AMUWEC20211912).

## Results

### Elevated TIM3 in macrophages is positively associated with chronic NASH fibrosis development in mice

**Fig. 1:**
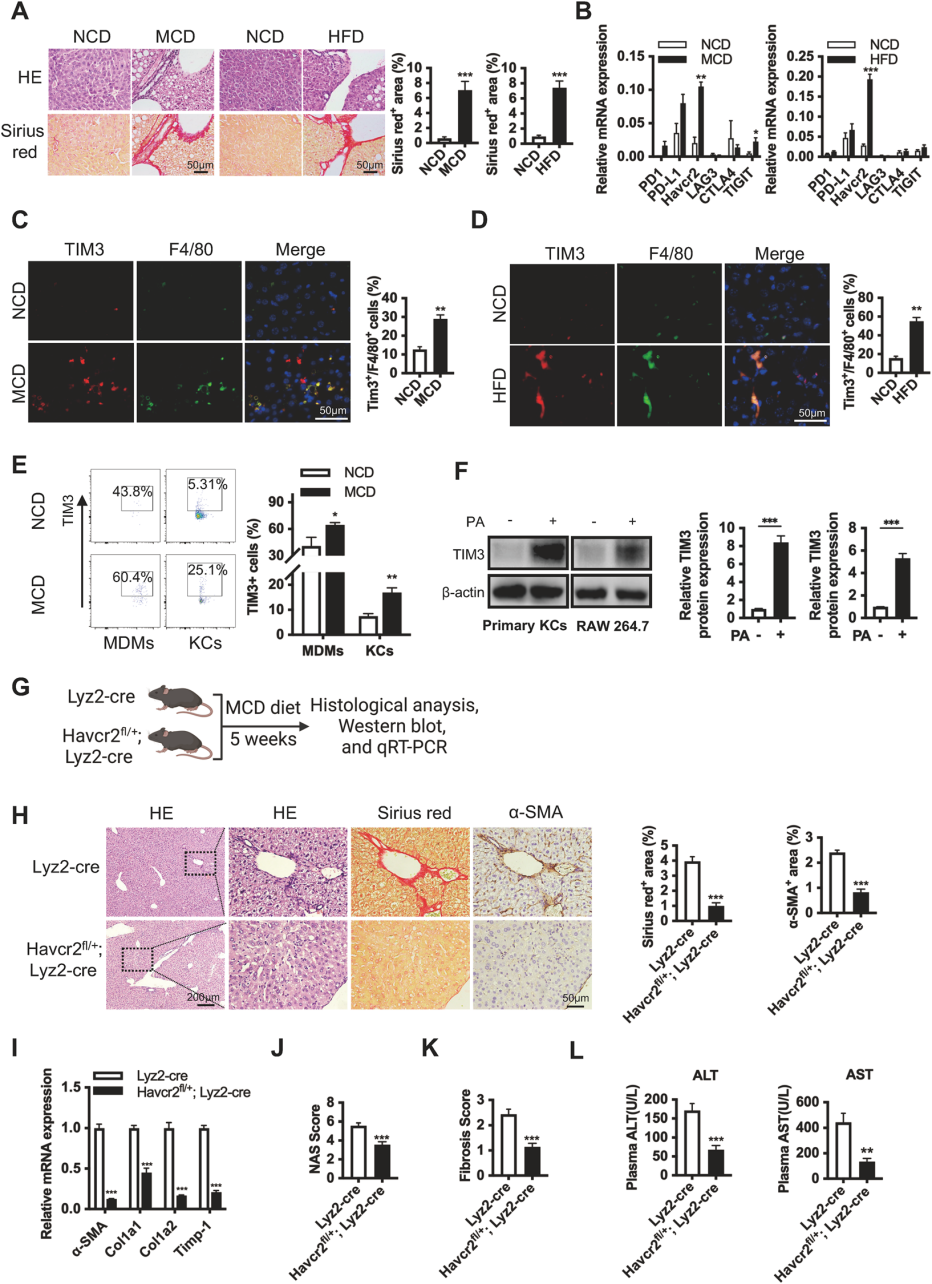
Elevated TIM3 in macrophages is positively associated with NASH fibrosis development in mice. **A** Representative HE- and Sirius red-stained sections from liver tissue of mice fed a negative control diet (NCD) and methionine and choline-deficient (MCD) diet for 5 weeks, and NCD and high-fat diet (HFD) for 26 weeks (*n* = 6/group). Quantification of Sirius red-positive areas from 6 regions. **B** qPCR was used to measure the mRNA expression levels of the immune checkpoint molecules PD-1, PD-L1, Havcr2, LAG3, CTLA4, and TIGIT from the liver tissue of mice in **A**. **C**, **D** Representative immunofluorescence staining sections from liver tissue of mice in **A**. Quantification of TIM3-positive F4/80-positive cells. **E** Isolated hepatic F4/80^high^CD11b^low^ Kupffer cells (KCs) and F4/80^low^CD11b^high^ monocyte-derived macrophages (MDMs), from mice treated with NCD and MCD for 5 weeks were analyzed for TIM3 expression by flow cytometry. Quantification of the percentage of TIM3-positive cells (*n* = 6/group). **F** TIM3 protein expression levels in primary KCs isolated from NCD-fed WT mice and RAW264.7 cells treated with 0.5 mM palmitic acid (PA) for 24 h. The relative protein expression was normalized to the level of β-actin. **G**–**L** Myeloid Havcr2 knockdown (Havcr2^fl/+^; Lyz2-cre) mice and Lyz2-cre mice were fed an MCD diet for 5 weeks (*n* = 7/group). IHC staining and Sirius red staining showed fibrosis in WT and Havcr2 knockdown mice (**H**). The mRNA expression levels of α-SMA, Col1a1, Col1a2, and Timp-1 in liver tissues of mice were determined by qPCR (**I**). The NAFLD activity score (NAS) and fibrosis score of liver tissues were quantified (**J**, **K**). The serum ALT and AST levels are shown in **L**. Data are presented as the mean ± SEM; significance determined by Student’s unpaired *t*-test (**A**, **C**, **D**, **F**, **H**, **J**–**L**) and two-way ANOVA with Bonferroni’s multiple comparisons test (**B**, **E**, **I**). *p < 0.05, **p < 0.01, ***p < 0.001.

To evaluate the fibrotic immune microenvironment in chronic NASH, we first established NASH fibrosis mice after long-term MCD or HFD feeding. Liver fibrosis was observed after 5 weeks of MCD or 26 weeks of HFD feeding, as indicated by Sirius red staining and high expression of fibrosis-associated biomarkers (α-SMA, Col1a1, Col1a2, PDGFβ, and Timp-1) (Fig. 1A, S1A, B), while acute NASH was induced in 2-week MCD-fed mice, as indicated by high expression of inflammation-associated factors (IL-1β, IL-6, and TNF-α) (Figure S1C). The NAFLD activity score (NAS) and fibrosis score further demonstrated aggressive liver fibrosis in the MCD/HFD-fed mice (Fig. S1D). Then, we found that Havcr2 (encoding TIM3) was dramatically upregulated in fibrotic liver tissues compared with other checkpoint markers (PD1, PD-L1, LAG3, CTLA4, and TIGIT) in these liver tissues, suggesting that TIM3 may be involved in NASH fibrosis (Fig. 1B). IHC and IF staining showed that TIM3 was highly expressed in macrophages (F4/80^+^) in the MCD/HFD group compared with the NCD group (Fig. 1C, D, S1E). Subsequently, we confirmed that TIM3-positive macrophages (especially KCs) were accumulated in a time-dependent course in MCD-fed mice (Fig. 1E, S1F). Furthermore, the Havcr2 expression in primary KCs was significantly higher in the MCD group (Fig. S1G). As expected, TIM3 expression was increased in primary KCs and RAW264.7 cells after palmitic acid (PA) treatment, which indicated that fat accumulation stimulates TIM3 expression in macrophages (Fig. 1F, S1H).

To further explore the role of TIM3 in macrophages at different stages of NAFLD, we investigated the effect of Havcr2 haploinsufficiency on both 2 weeks of the early stage of NAFLD and 5 weeks of a later stage of NAFLD in Havcr2^fl/+^; Lyz2-cre or Havcr2 KD mice (Fig. 1G, S2A, B, H). Knockdown of Havcr2 specifically in the macrophages was achieved under the control of the lysozyme M (encoded by the Lyz2 gene) promoter [10]. Havcr2^fl/+^; Lyz2-cre mice fed an MCD diet for 2 weeks showed a significant increase of proinflammatory cytokines compared with Lyz2-cre mice, with no significant difference in fibrosis (Fig. S2I, J). This finding is consistent with previous study that TIM3 played a protective role in liver injury induced by short-term MCD feeding [9]. Liver fibrosis was significantly attenuated after Havcr2 haploinsufficiency in 5-week MCD-fed mice as assessed by Sirius red staining and α-SMA IHC staining and as indicated by the expression levels of fibrosis-related markers, but the expression of proinflammatory cytokines was not significantly changed (Fig. 1H, I, S2C–E, K, L). The NASH and liver fibrosis score also showed that Havcr2 knockdown mice had lower levels of NASH fibrosis than wild-type mice (Fig. 1J, K, S2F, M). In addition, liver function was recovered after Havcr2 haploinsufficiency in 5-week MCD-fed mice (Fig. 1L, S2G). Taken together, these data demonstrate that elevated TIM3 in macrophages is associated with chronic NASH fibrosis development.

### β-TrCP promotes the ubiquitinated TIM3 membrane localization and then binds with PI3K to induce M2 polarization

**Fig. 2:**
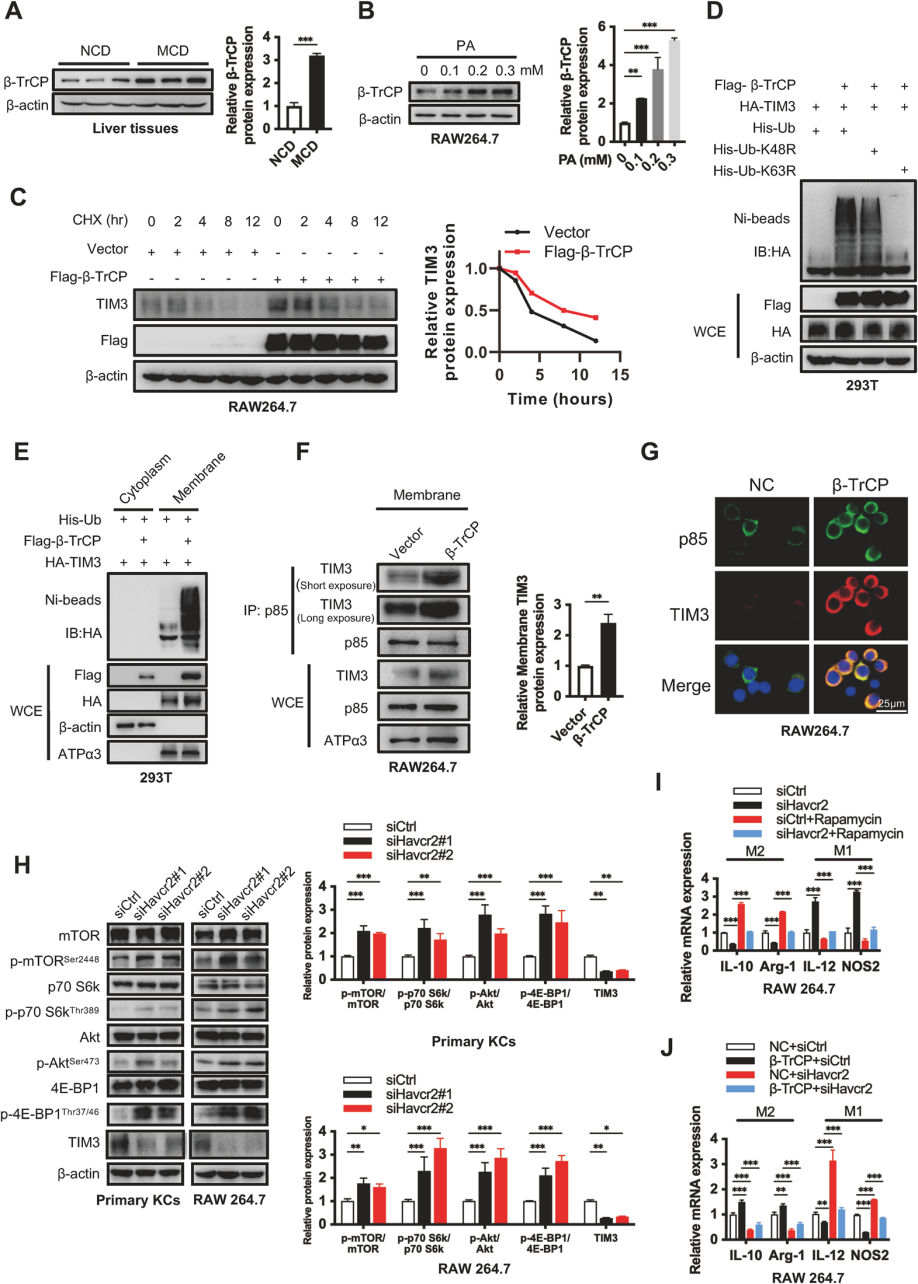
β-TrCP promotes TIM3 ubiquitination and transmembrane localization, leading to M2 polarization via inhibition of the PI3K/mTOR pathway in macrophages. **A** β-TrCP protein expression levels in NCD or MCD-fed mice (*n* = 3/group). The relative protein expression was normalized to the level of β-actin. **B** β-TrCP protein expression levels in RAW264.7 cells treated with PA for 24 h. The relative protein expression was normalized to the level of β-actin. **C** RAW264.7 cells were transfected with indicated plasmids for 48 h and then treated with CHX (100 μg/mL) for the indicated time intervals. The immunoblot results were quantified using the Image J software and normalized to the Vector group. **D** β-TrCP mediated K63-linked polyubiquitination of TIM3. HEK293T cells were transfected with the β-TrCP plasmids along with WT-ubiquitin or its mutants (K63R and K48R) for 72 h, and immunoprecipitation with Ni-beads and analyzed by Western blot. **E** Ubiquitylated TIM3 by β-TrCP was on the surface of the membrane. HEK293T were transfected with indicated plasmids for 72 h, and cytoplasm and membrane proteins were respectively subjected to immunoprecipitation with Ni-beads and analyzed by western blot. **F**, **G** Interaction of TIM3 with PI3K p85 was analyzed by co-IP or immunofluorescence staining in RAW264.7 cells treated with or without β-TrCP plasmids. The membrane TIM3 protein interaction with PI3K p85 was normalized to the level of ATPα3. **H**, **I** Primary KCs were transfected a nontargeting siRNA control (siCtrl) or siRNA targeting Havcr2 (siHavcr2) with or without the mTOR inhibitor rapamycin (100 nM) for 48 h. The effect of siHavcr2 and AKT/mTOR pathway were detected by western blot. The relative protein expression was normalized to the level of β-actin (**H**). Macrophage polarization markers were analyzed by qPCR (**I**) (*n* = 3/group). **J** RAW264.7 cells were transfected with β-TrCP plasmid or siHavcr2 for 48 h. Macrophage polarization markers at the mRNA level were detected by qPCR (*n* = 3/group). Data are presented as the mean ± SEM; Student’s unpaired *t*-test was used in **A** and **F**; one-way ANOVA was used in **B**; two-way ANOVA was used in **H**–**J**. *p < 0.05, **p < 0.01, ***p < 0.001.

TIM3, a transmembrane protein expressed on the surface of immune cells or tumor cells, has been associated with both inhibitory and co-stimulatory functions, depending in part on the specific cell type and immune response course. However, how TIM3 is translocated to the cell membrane remains unknown. Ubiquitination plays a key role in the regulation of protein degradation, activation, localization, and interaction with other cellular molecules [11, 12]. To clarify whether TIM3 activation requires ubiquitination modification, we examined the TIM3 protein sequence, and found an evolutionarily conserved consensus binding motif (DSGXT) for β-TrCP at codons 194–198 (DSGXT) of human TIM3 (Fig. S3A), suggesting that TIM3 may be a substrate of β-TrCP. We found that β-TrCP expression was increased in MCD-fed mice and PA-treated RAW264.7 cells (Fig. 2A, B). Overexpression of β-TrCP extended the half-life of TIM3 (Fig. 2C). Significantly, β-TrCP could promote K63-linked TIM3 ubiquitination, and this ubiquitinated TIM3 translocated to the cell membrane (Fig. 2D, E). Furthermore, β-TrCP promoted TIM3 binding with p85 on the surface of macrophages (Fig. 2F, G). A previous study reported that TIM3 could compete with PI3K p110 to bind p85, inhibiting downstream Akt/mTORC1 signaling in NK cells [13]. The mTOR signaling pathway can promote M1 macrophage polarization [14, 15]. Considering the critical role of macrophage phenotypic changes in the development of NASH fibrosis [16, 17], we hypothesized that TIM3 may promote the M2 polarization of macrophages by binding with PI3K and inhibiting the mTOR pathway. As expected, endogenous p85 was pulled down by endogenous TIM3 in RAW264.7 cells (Fig. S3B). Reciprocally, endogenous TIM3 was pulled down by p85 in RAW264.7 cells (Fig. S3C). Havcr2 knockdown upregulated p-mTOR and its downstream molecules p-4E-BP1 and p-p70 S6k in primary KCs and RAW264.7 cells and promoted M1 polarization (Fig. 2H, I). This action was significantly blocked by rapamycin, an mTOR inhibitor (Fig. 2I). In addition, we found that Havcr2 knockdown significantly attenuated β-TrCP-mediated M2 macrophage polarization in RAW264.7 cells (Fig. 2J). 2-week MCD-fed mice primarily enhanced M1 polarization and liver injury, while 5-week MCD-fed mice mainly increased M2 polarization and NASH fibrosis (Fig. S1B, C, S3D). Havcr2 haploinsufficiency further enhanced macrophage M1 polarization in 2-week MCD-fed mice, leading to liver injury, and attenuated macrophage M2 polarization in 5-week MCD-fed mice, protecting the liver from NASH fibrosis (Fig. S3E). Together, these results suggest that high fat induces β-TrCP high expression, which promotes TIM3 polyubiquitination and transmembrane localization, leading to M2 polarization via TIM3-PI3K interaction.

### Elevated TIM3 is attributed to TBP expression in macrophages

**Fig. 3:**
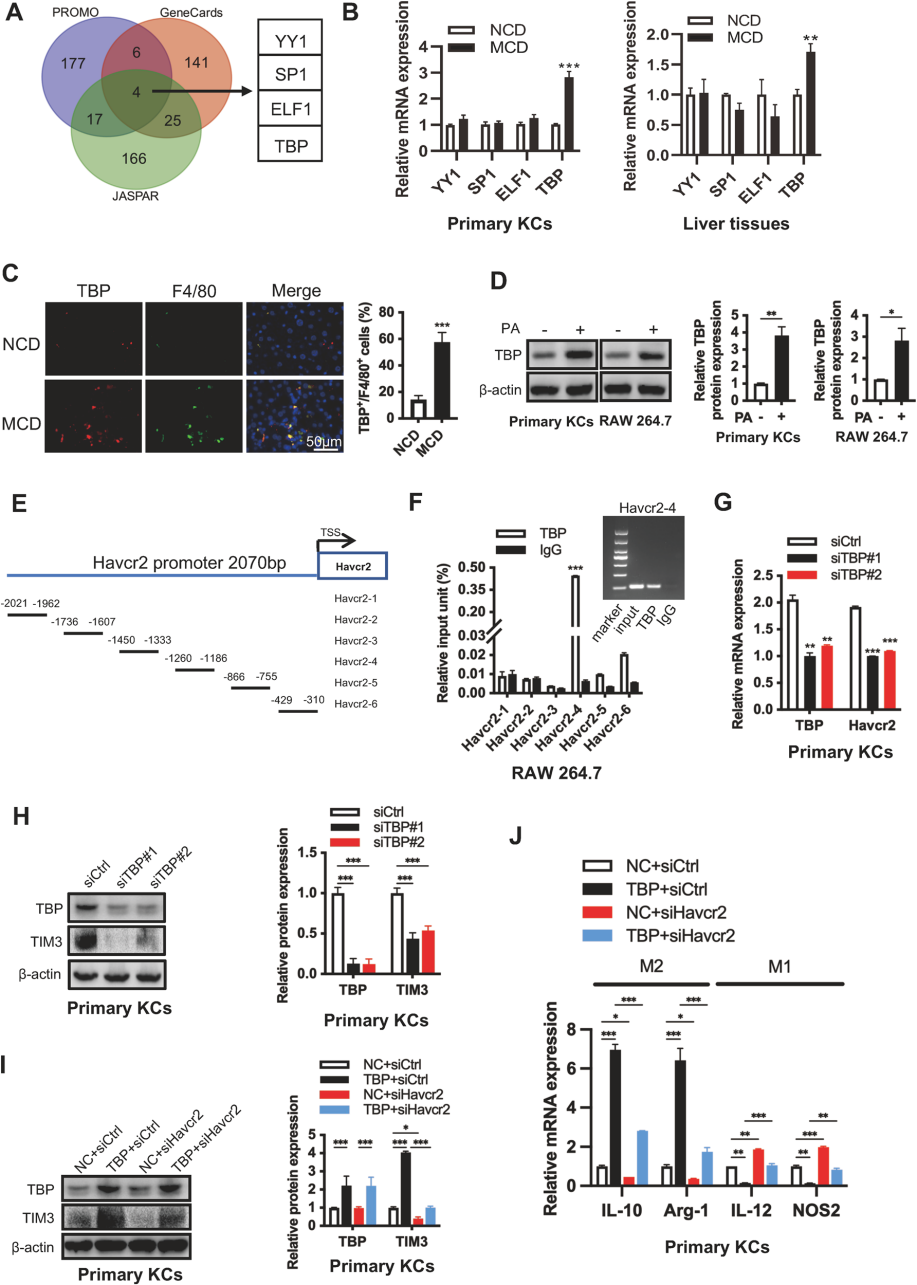
Elevated TIM3 is attributed to TBP high expression in macrophages. **A** Venn diagram showing potential upstream transcription factors of Havcr2 predicted by the PROMO, GeneCards, and JASPAR databases. **B** Relative mRNA levels of YY1, SP1, ELF1, and TBP were detected in liver tissues and primary KCs isolated from NCD-fed or MCD-fed mice (*n* = 6/group). **C** Representative immunofluorescence staining showing TBP (red) and F4/80 (green) expression in liver tissue. Quantification of the percentage of TBP-positive cells in 100 F4/80-positive cells. **D** TBP protein expression levels in primary NCD-KCs and RAW264.7 cells after exposure to 0.5 mM PA for 24 h. The relative protein expression was normalized to the level of β-actin. **E** A schematic illustration of the six primer positions designed in the Havcr2 promoter sequence for possible TBP binding sites. **F** RAW264.7 cells were used in the ChIP assay, and DNA-protein complexes were obtained and incubated with anti-IgG or anti-TBP antibodies. Enriched DNAs were used for qPCR and agarose gel electrophoresis assays using the primers presented in **E**. **G**, **H** Primary KCs isolated from MCD-fed WT mice were transfected with a nontargeting siRNA control (siCtrl) or siRNA targeting TBP (siTBP) for 48 h. The expression of TIM3 and TBP was examined by qPCR (**G**) and western blot (**H**). The relative protein expression was normalized to the level of β-actin (**H**). (**I**, **J**) Primary KCs isolated from MCD-fed WT mice were transfected with TBP plasmid or siHavcr2 for 48 h. Havcr2 and TBP at the protein levels were detected by western blot (**I**). The relative protein expression was normalized to the level of β-actin (**I**). Macrophage polarization markers at the mRNA level were detected by qPCR (**J**) (*n* = 3/group). Data are presented as the mean ± SEM; Student’s unpaired *t*-test was used in **C** and **D**; two-way ANOVA with Bonferroni’s multiple comparisons test was used in **B**, **F**, **G**–**J**. Unpaired t-test was used in **C**. *p < 0.05, **p < 0.01, ***p < 0.001.

To clarify how macrophage TIM3 expression was increased during NASH fibrosis, three online databases (PROMO, GeneCards, and JASPAR) were used to predict the potential upstream transcription factors of TIM3 (Fig. 3A). There are four transcription factors (YY1, SP1, ELF1, and TBP) that may control TIM3 expression, in which the expression of TBP but not YY1, SP1, or ELF1 was significantly increased in MCD-treated primary KCs and liver tissues (Fig. 3B, C). We also confirmed that fat accumulation stimulated TBP expression in macrophages (Fig. 3D, S4A). To validate TBP binding to the Havcr2 promoter, the top six scoring sites were selected and designed for the ChIP assay (Fig. 3E). We found that TBP binds to Havcr2 in the region between -1260 and -1186 bp (Fig. 3F). Knockdown of TBP significantly decreased the mRNA and protein levels of TIM3, leading to activation of AKT/mTOR signaling and M1 macrophage polarization in both primary KCs and RAW264.7 cells (Fig. 3G, H, S4B–F). Reciprocally, overexpression of TBP increased TIM3 expression at both the mRNA and protein levels, promoted M2 polarization via inactivation of AKT/mTOR signaling (Fig. 3I, J, S4G–J). Taken together, elevated TIM3 in macrophages depends on TBP.

### Downregulation of miR-4524a-5p increases TBP and TIM3 expression in macrophages

**Fig. 4:**
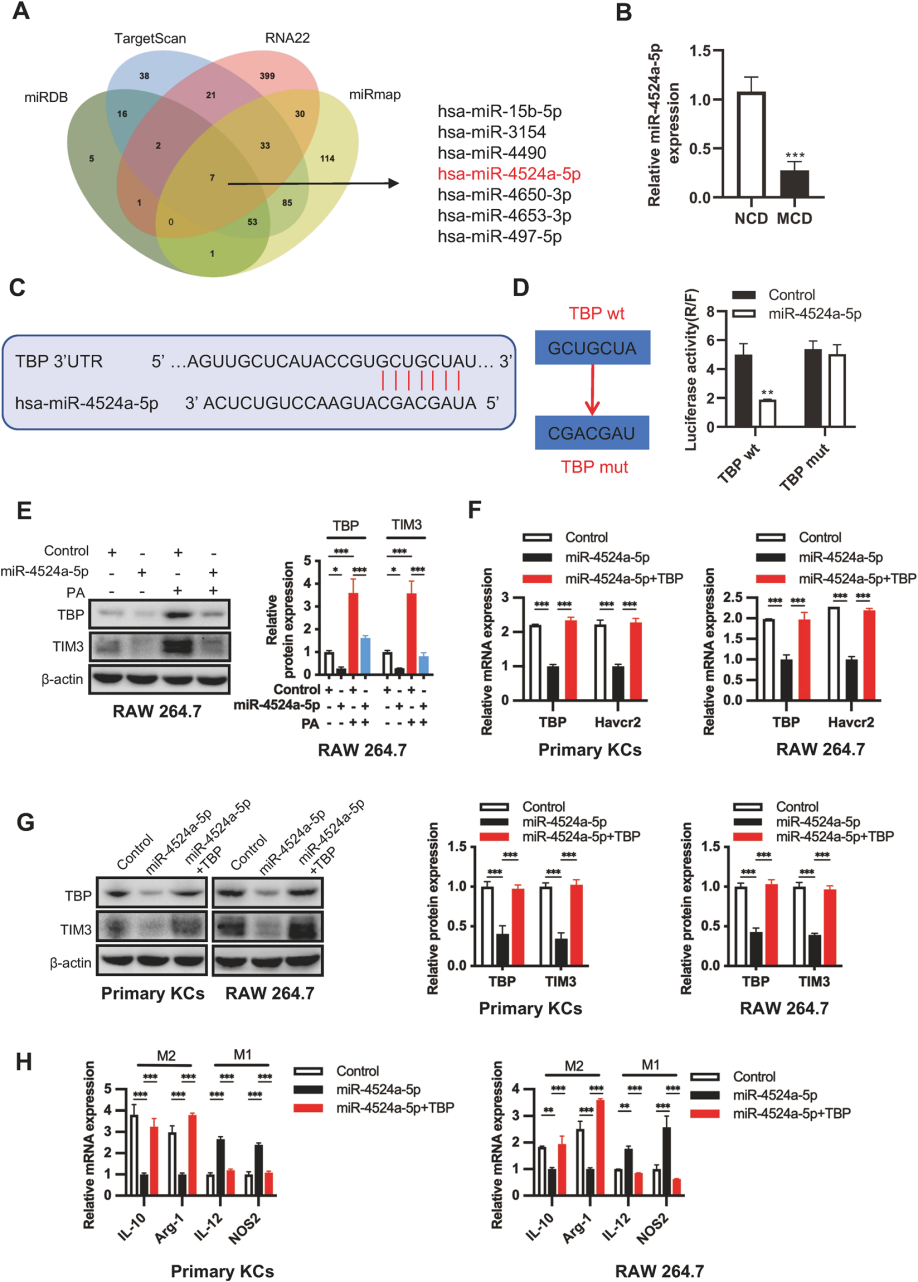
Downregulation of miR-4524a-5p increases TBP and TIM3 expression in macrophages and induces M1 macrophage polarization into M2 macrophages. **A** Venn diagram showing potential microRNAs targeting TBP predicted by the miRDB, TargetScan, RNA22, and miRmap databases. **B** Relative expression levels of miR-4524a-5p were detected by qPCR in liver tissues from 5-week NCD-fed or MCD-fed mice (*n* = 6/group). **C** The TargetScan database was used to analyze the possible binding site of miR-4524a-5p and the TBP-3’-UTR. **D** A luciferase reporter gene assay was performed in RAW264.7 cells transfected with miR-4524a-5p or control and wt-TBP-3’-UTR (TBP wt) or mut-TBP-3’-UTR (TBP mut) plasmids. **E** RAW264.7 cells were transfected with miR-4524a-5p or control for 48 h and then treated with or without PA (0.5 mM) for 24 h. The protein expression levels of TBP and TIM3 in whole cell lysates were determined by western blot. The relative protein expression was normalized to the level of β-actin. **F**–**H** Primary KCs isolated from MCD-fed WT mice and RAW264.7 cells were transfected miR-4524a-5p with or without the TBP overexpression plasmid for 48 h. The mRNA expression levels of Havcr2, TBP, and macrophage polarization markers in whole cell lysates were detected by qPCR (**F**, **H**). The protein expression of TIM3 and TBP was detected by western blot (**G**) (*n* = 3/group). The relative protein expression was normalized to the level of β-actin (**G**). Data are presented as the mean ± SEM; significance is determined by Student’s two-tailed t-test (**B**) and two-way ANOVA with Bonferroni’s multiple comparisons test (**D**–**H**). *p < 0.05, **p < 0.01, ***p < 0.001.

We next aimed to determine the cause of TBP overexpression during NASH fibrosis. Given the critical role of miRNA in regulating the expression level of transcription factors, we predicted miRNAs that could possibly bind to the 3’-UTR of TBP by analyzing databases (miRDB, TargetScan, RNA22, and miRmap). A total of seven miRNAs (miR-15b-5p, miR-3154, miR-4490, miR-4524a-5p, miR-4650-3p, miR-4653-3p, and miR-497-5p) that appeared in all four databases were selected as target miRNAs (Fig. 4A). Considering that the development of fibrosis in the liver is closely related to the occurrence of HCC, we performed the expression levels and Kaplan‒Meier analysis of these seven miRNAs in HCC patient samples in the TCGA database. Among them, miR-4524a-5p were downregulated in HCC tissues, and patients with lower miR-4524a-5p expression had a poorer OS (Fig. S5A, B). We found that miR-4524a-5p had lower expression levels in liver tissues of MCD-fed mice than NCD-fed mice (Fig. 4B). To explore the regulatory role of miR-4524a-5p on TBP, we predicted the possible binding regions of miR-4524a-5p with the 3’-UTR of TBP by database analysis and confirmed by a luciferase reporter gene assay (Fig. 4C, D). As expected, the TBP expression in the presence or absence of PA treatment was significantly decreased after overexpression of miR-4524a-5p in RAW264.7 cells (Fig. 4E). Meanwhile, overexpression of miR-4524a-5p mimics significantly reduced TBP and TIM3 expression and induced M1 macrophage polarization in macrophages (Fig. 4F–H). These actions were attenuated by overexpression of TBP (Fig. 4F–H). The results demonstrated that decreased expression of miR-4524a-5p in NASH fibrosis attenuates its interaction with TBP, increases TIM3 expression, and then induces M2 macrophage polarization.

### miR-4524a-5p/TBP/TIM3/mTOR signaling pathway promotes HSC activation by increasing TGF-β expression in macrophages

**Fig. 5:**
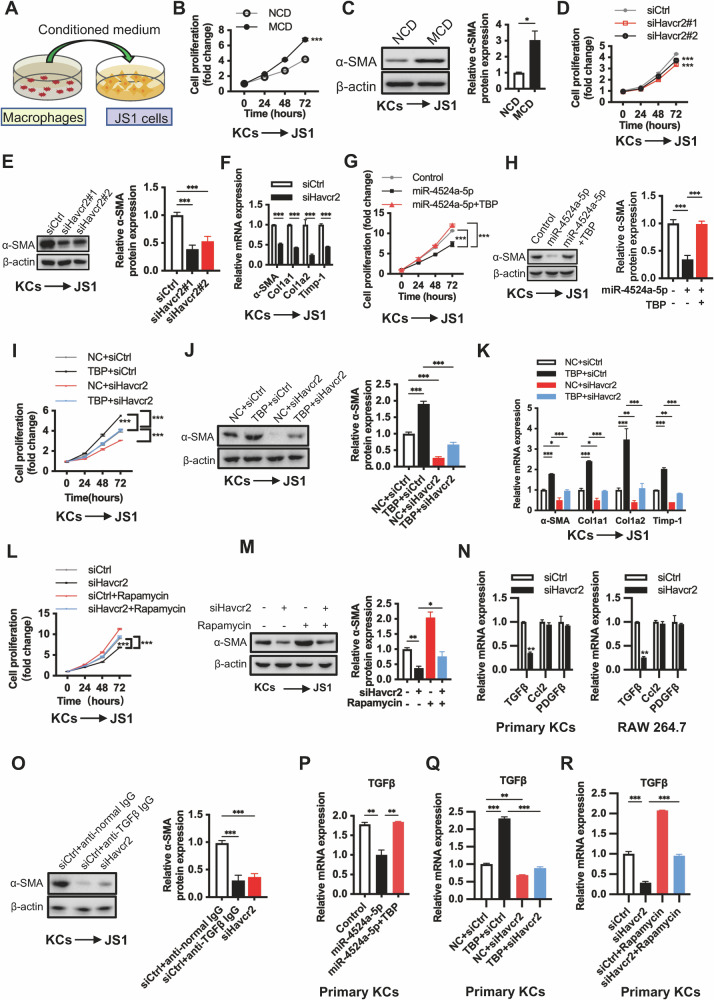
miR-4524a-5p/TBP/TIM3/mTOR signaling pathway promotes HSC activation by increasing TGF-β expression in macrophages. **A** A schematic diagram showing the methodology used to study the effect of macrophages on hepatic stellate cell activation. Conditioned medium (CM) from the treated primary KCs and RAW264.7 cells was added to JS1 cells. **B**, **C** Primary KCs isolated from mice fed NCD and MCD for 5 weeks were cultured for 24 h. The CM was harvested and added to mouse JS1 cells (*n* = 3/group). Cell proliferation of JS1 cells was assessed by CCK-8 assay (**B**). The protein expression level of α-SMA was used to assess the activation of JS1 cells (**C**). The relative protein expression was normalized to the level of β-actin (**C**). **D**–**F** Primary KCs isolated from MCD-fed WT mice were treated with siCtrl or siHavcr2 for 48 h, and the CM was harvested and added to mouse JS1 cells. The JS1 cells were maintained in the CM, and at specified time points, cell proliferation was assessed by CCK-8 assay (**D**), and HSC activation and fibrosis-associated biomarkers were analyzed by western blot or qPCR (**E**, **F**) (*n* = 3/group). The relative protein expression was normalized to the level of β-actin (**E**). **G**, **H** Primary KCs isolated from MCD-fed WT mice were transfected with miR-4524a-5p with or without the TBP overexpression plasmid for 48 h, and the CM was harvested and added to mouse JS1 cells for different time intervals. JS1 cell proliferation was assessed by CCK-8 assay (**G**). α-SMA protein expression in whole cell lysates was determined by western blot (**H**) (*n* = 3/group). The relative protein expression was normalized to the level of β-actin (**H**). **I**–**K** Primary KCs isolated from MCD-fed WT mice were transfected TBP plasmid with or without siHavcr2 for 48 h. The CM was collected and added to JS1 cells. Cell proliferation was assessed by CCK-8 assay (**I**). The protein level of α-SMA in JS1 cells was determined by western blot (**J**), and the mRNA expression levels of α-SMA, Col1a1, Col1a2, and Timp-1 in JS1 cells were measured by qPCR (**K**) (*n* = 3/group). The relative protein expression was normalized to the level of β-actin (**J**). **L**, **M** Primary KCs isolated from MCD-fed WT mice were transfected siHavcr2 with or without the mTOR inhibitor rapamycin (100 nM) for 48 h, and the CM was collected and added to JS1 cells. Cell proliferation was assessed by CCK-8 assay (**L**). α-SMA protein expression in whole cell lysates was determined by western blot (**M**) (*n* = 3/group). The relative protein expression was normalized to the level of β-actin (**M**). **N** qPCR was used to measure profibrotic factor (TGF-β, Ccl2, and PDGF-β) expression levels in primary KCs isolated from MCD-fed WT mice and RAW264.7 cells treated with siHavcr2 for 48 h (*n* = 3/group). **O** Primary KCs were treated with anti-TGF-β antibody or siHavcr2 for 48 h, followed by the addition of CM to JS1 cells for 24 h (*n* = 3/group). The relative protein expression was normalized to the level of β-actin. **P**–**R** qPCR was used to measure TGF-β expression levels in primary KCs treated as described in **G**, **I** and **L** (*n* = 3/group). Data are presented as the mean ± SEM; Student’s unpaired t-test was used in **C**; one-way ANOVA with Bonferroni’s multiple comparisons test was used in (**E**, **H**, **J**, **M**, and **O–R**); two-way ANOVA with Bonferroni’s multiple comparisons test was used in **B**, **D**, **F**, **G**, **I**, **K**, **L**, and **N**. *p < 0.05, **p < 0.01, ***p < 0.001.

We next explored how macrophages contribute to fibrosis progression, focusing on the communication between macrophages and HSCs. As shown in Fig. 5A, conditioned medium (CM) from primary KCs and RAW264.7 cells was added to the mouse hepatic stellate cell line JS1. We found that CM promoted the proliferation and activation of JS1 cells (Fig. 5B, C). Reciprocally, the CM from Havcr2 knockdown macrophages effectively inhibited HSC proliferation and activation (Fig. 5D–F, S6A, B). These findings indicated that high expression of TIM3 in macrophages promoted HSC proliferation and activation through cell-to-cell interactions. Increasing miR-4524a-5p attenuated the effect of macrophages on JS1 cell proliferation and activation, while overexpression of TBP abolished the inhibitory effect of miR-4524a-5p (Fig. 5G, H, S6C, D). CM from TBP-knockdown macrophages inhibited cell proliferation and reduced α-SMA in JS1 cells (Fig. S6E, F). Furthermore, CM from TBP-overexpressing macrophages promoted HSC proliferation and activation, and knockdown of Havcr2 significantly blocked this effect (Fig. 5I–K, S6G–I). CM from rapamycin-treated KCs dramatically attenuated Havcr2-knockdown-mediated and TBP-knockdown-mediated inhibition of HSC proliferation and activation (Fig. 5L, M, S6J–M). These results demonstrated that the macrophage miR-4524a-5p/TBP/TIM3/mTOR signaling pathway promotes HSC activation.

To determine whether macrophages release factors that activate HSCs during NASH fibrosis, we found that Havcr2 knockdown significantly reduced TGF-β but not PDGFβ or Ccl2 in macrophages (Fig. 5N), suggesting that TGF-β may be involved in HSC activation and NASH fibrosis. We confirmed that CM from anti-TGF-β-treated KCs and RAW264.7 cells do not activate HSCs (Fig. 5O, S7A). Furthermore, miR-4524a-5p inhibited the production of TGF-β, and overexpression of TBP attenuated this action in macrophages (Fig. 5P, S7B). Overexpression of TBP promoted TGF-β upregulation, and Havcr2 knockdown blocked this effect (Fig. 5Q, S7C). Inhibition of mTOR by rapamycin reversed Havcr2/TBP knockdown-mediated downregulation of TGF-β production (Fig. 5R, S7D). These results suggest that miR-4524a-5p/TBP/TIM3/mTOR signaling induces TGF-β secretion in macrophages, which promotes HSC proliferation and activation.

### Blockade of TIM3 induces M1 macrophage polarization and prevents chronic NASH fibrosis in mice

**Fig. 6:**
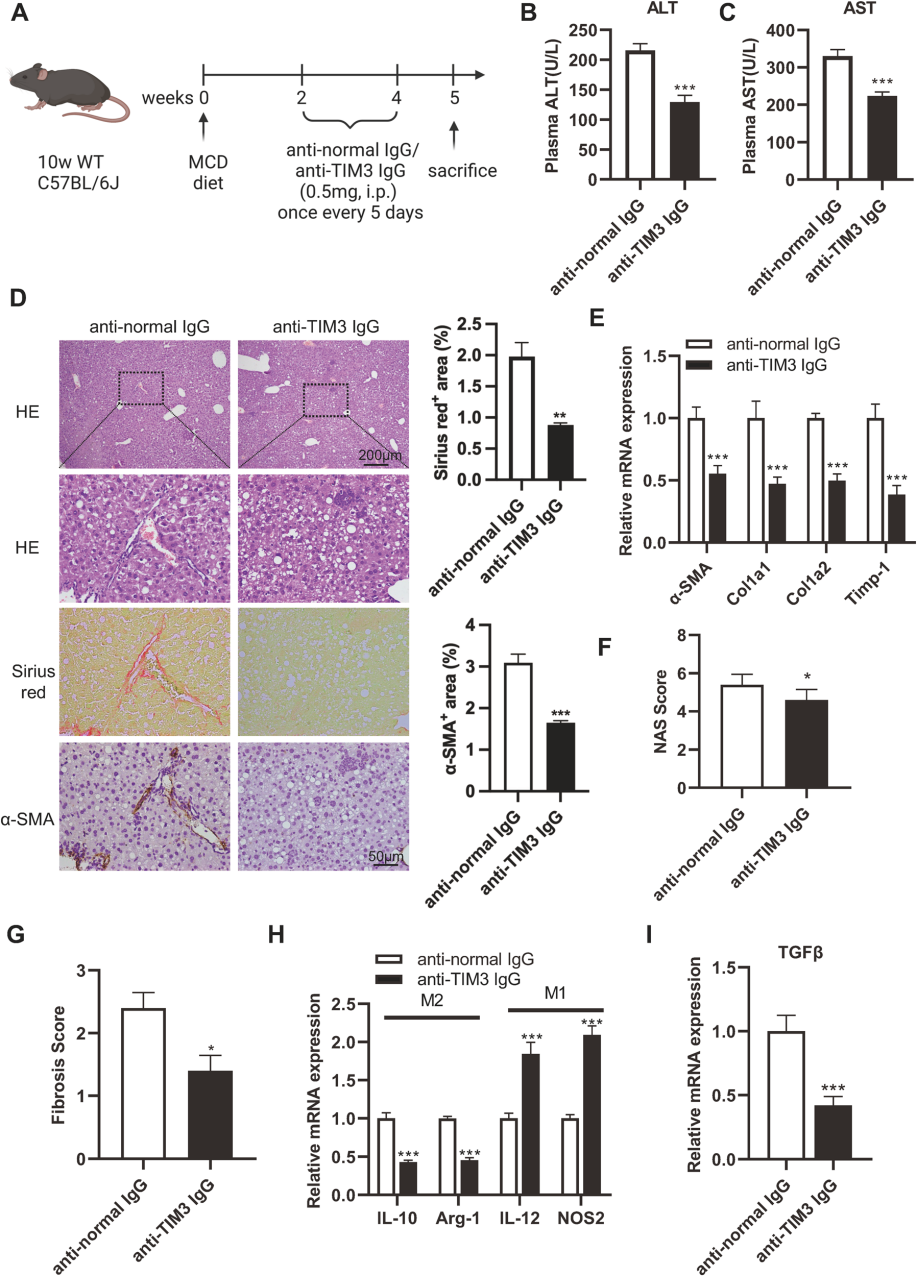
Blockade of TIM3 induces M1 macrophage polarization and prevents NASH fibrosis in mice. **A** Experimental scheme of C57BL/6 mice fed an MCD diet that were intraperitoneally injected with anti-TIM3 IgG or normal IgG once every five days. **B**, **C** Low serum ALT and AST levels in the anti-TIM3 group were found in mice fed an MCD diet for 5 weeks (*n* = 5/group). **D** Representative HE, Sirius red, and α-SMA IHC-stained sections from the indicated groups. Quantification of Sirius red- and α-SMA-positive areas. **E** The mRNA expression levels of α-SMA, Col1a1, Col1a2, and Timp-1 were determined by qPCR. **F, G** The NAS and fibrosis score of liver tissues were analyzed. qPCR was used to measure macrophage polarization markers (**H**) and TGF-β (**I**) at the mRNA expression level in the normal IgG and anti-TIM3 IgG groups. Data are presented as the mean ± SEM; Student’s unpaired *t-*test was used in **B**–**D**, **F**, **G**, and **I**; two-way ANOVA with Bonferroni’s multiple comparisons test was used in E and H. *p < 0.05, **p < 0.01, ***p < 0.001.

To elucidate the impacts of TIM3-regulated macrophages on chronic NASH fibrosis, we evaluated the effect of anti-TIM3 treatment on NASH fibrosis. We intraperitoneally injected anti-TIM3 antibody or normal control into MCD-fed C57BL/6J mice (Fig. 6A). The anti-TIM3-treated mice had lower plasma ALT and AST levels than the control mice, while body weight, liver-to-body weight ratio, plasma cholesterol, and plasma triglycerides were not significantly different between the two groups (Fig. 6B, C, S8A–D). Crucially, TIM3 blockade retarded HSC activation and fibrosis during NASH development, as indicated by Sirius red staining and fibrosis-related biomarkers (Fig. 6D, E, S8E). Compared with the control group, the anti-TIM3-treated group had a lower NASH and liver fibrosis score (Fig. 6F, G, S8F). These data indicated that anti-TIM3 treatment recovered liver function and attenuated fibrosis in NASH development. Meanwhile, anti-TIM3 treatment promoted M1 macrophage polarization and suppressed the secretion of TGF-β by profibrotic M2 polarized macrophages (Fig. 6H, I). Taken together, our results suggest that targeting TIM3 effectively promotes M1 macrophage polarization to reduce TGF-β secretion, leading to inactivation of HSCs and inhibition of fibrosis in NASH development.

### Elevated TIM3 expression is positively correlated with TBP in patients with late-stage liver fibrosis

**Fig. 7:**
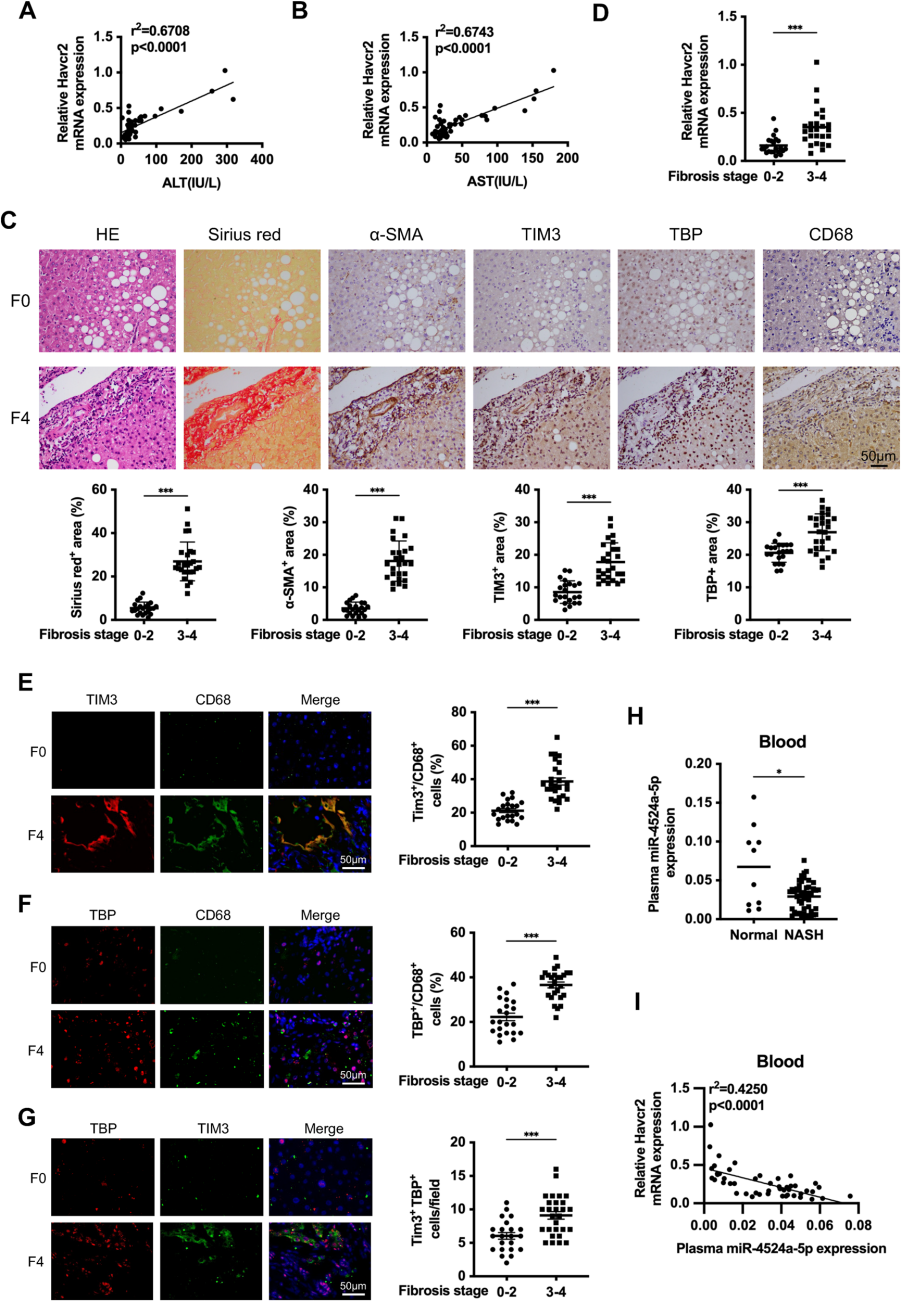
Elevated TIM3 expression is negative correlated with plasma miR-4524a-5p and positively correlated with TBP in NASH patients with late-stage liver fibrosis. **A**, **B** Correlation between liver Havcr2 mRNA expression and plasma ALT and AST levels in NASH patients (*n* = 48). **C** Representative HE, Sirius red and α-SMA, TIM3, TBP, and CD68 IHC-stained sections from the liver tissue of NASH patients with fibrosis stage F0-2 (*n* = 22) and fibrosis stage F3-4 (*n* = 26). Quantification of Sirius red-, α-SMA-, TIM3-, and TBP-positive areas from 6 regions. **D** The Havcr2 mRNA levels at fibrosis stages F0-2 and F3-4 were quantified in 48 NASH patients. **E**, **F** Representative sections with immunofluorescence staining showing the expression of TIM3 or TBP (red) at CD68 (green) in NASH patients at fibrosis stage F0 or F4. Quantification of TBP/TIM3-positive cells among more than 100 CD68-positive cells. **G** Representative sections with immunofluorescence staining showing the expression of TIM3 (green) and TBP (red) in NASH patients at fibrosis stage F0 or F4. Quantification of TIM3/TBP-positive cells in more than 100 cells. **H** The plasma miR-4524a-5p expression levels were quantified in 48 NASH patients and 10 healthy donors. **I** Correlation between liver Havcr2 mRNA expression and plasma miR-4524a-5p expression levels in NASH patients (*n* = 48). Data are presented as the mean ± SEM; significance determined by Welch’s two-tailed t-test in **C**–**H**. *p < 0.05, ***p < 0.001.

To assess the clinical relevance of our findings, we examined the expression of Havcr2 in liver tissues from 48 NASH patients. Pearson correlation analysis showed that plasma ALT and AST levels were positively correlated with Havcr2 expression in NASH patients (Fig. 7A, B). We then divided human NASH specimens into two groups by HE, Sirius red, and α-SMA IHC staining: stage F0-2 (no or slight fibrosis) and stage F3-4 (massive fibrosis). TIM3 was higher in NASH patients with stage F3-4 disease than in those with stage F0-2 disease (Fig. 7C, D). IF staining further revealed that TIM3- and TBP-positive macrophages were abundant in patients with stage F3-4 disease but not in those with stage F0-2 disease (Fig. 7E–G). These results suggest a positive correlation between TIM3 and TBP in macrophages in the late stage of human NASH fibrosis. Cells secrete miRNA into blood, saliva, urine, and other body fluids, providing a more convenient way for miRNA detection and application in disease diagnosis [18]. We detected and observed that the expression of miR-4524a-5p was lower in the blood of NASH patients compared with healthy donors (Fig. 7H). Moreover, the blood miR-4524a-5p expression was negative correlation with Havcr2 expression, providing blood miR-4524a-5p as a new biomarker for the NASH fibrosis patients suitable for anti-TIM3 therapy (Fig. 7I).

## Discussion

**Fig. 8:**
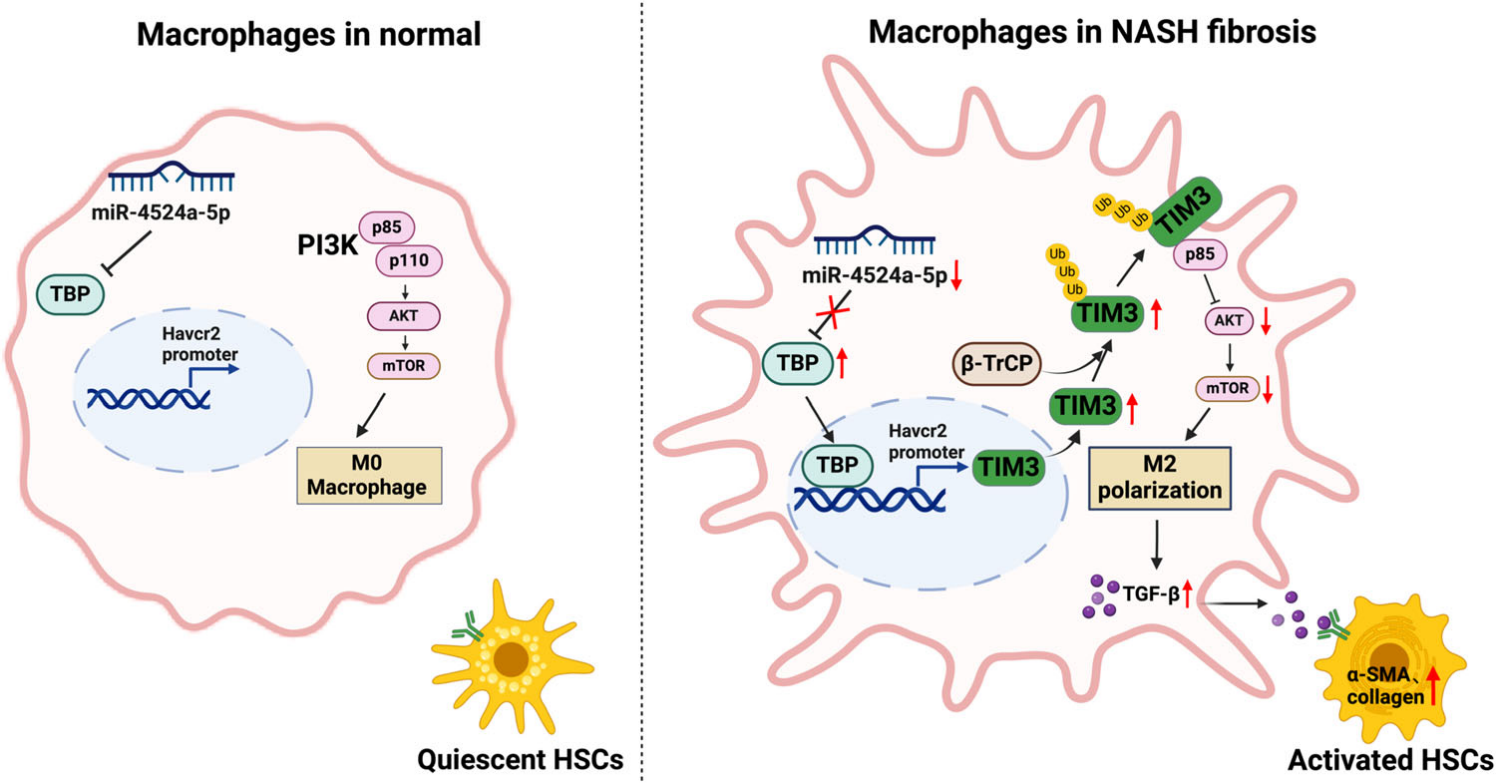
A graphical abstract of the role of macrophage TIM3 in NASH fibrosis. In normal liver, miR-4524a-5p in macrophages suppresses TBP/TIM3/PI3K/AKT/mTOR/TGFβ signaling, and HSCs remain quiescent. In fibrotic NASH, TBP/TIM3 signaling is restored by suppression of miR-4524a-5p. β-TrCP-mediated TIM3 ubiquitination and activation leads to PI3K/AKT/mTOR suppression and TGF-β induction, and the resulting increase and secretion of TGF-β from these macrophages activates HSCs.

The pathogenesis of liver fibrosis in NAFLD is not well understood, so there are currently no approved therapeutic agents. In the present study, we provided evidence that TIM3 expression was significantly increased in macrophages in mouse models of NASH fibrosis. We found that high fat upregulated TBP and β-TrCP, which induced membrane TIM3 polyubiquitination and activation, thereby promoting liver fibrosis. Our study showed that macrophage TIM3 knockdown or antibody blockade attenuated NASH-associated liver fibrosis in mice, providing a possible basis for immunotherapy of liver fibrosis (Fig. 8).

Immunotherapy is an emerging therapeutic method that can reverse the suppression of the immune response by blocking immune checkpoints, thereby eliminating cancer cells and inhibiting tumor growth [19]. As a new immune checkpoint molecule, TIM3 has received more attention, and several drugs targeting TIM3 have entered several phases of clinical trials [20, 21]. Multiple studies have shown the critical role of TIM3 in chronic viral infections, immune diseases, and cancer [22, 23]. However, the role of TIM3 in liver fibrosis is still unknown. Generally, macrophages are first polarized to pro-inflammatory M1 phenotype to assist the host against damages. Subsequently, macrophages are polarized to M2 phenotype to drive an anti-inflammatory response and to repair damaged tissues. Consistently, in our study, macrophages in 2-week MCD-fed mice were primarily polarized to M1 polarization, while macrophages in 5-week MCD-fed mice were mainly polarized to M2 phenotype. We found that knockdown of TIM3 in macrophages induced M1 polarization in vitro. In line with this finding, Havcr2 haploinsufficiency primarily enhanced macrophage M1 polarization in 2-week MCD-fed mice, which is consistent with previous study that the increased TIM3 protects MCD-fed mice from liver injury at the early stage [9], but Havcr2 knockdown attenuated macrophage M2 polarization in 5-week MCD-fed mice in vivo, leading to blocking liver NASH fibrosis. This is attributed to a large number of M2 macrophages recruited at the liver in the later stage of NASH. These findings indicate TIM3 as an anti-inflammatory role in the early stage of NASH and as a pro-fibrotic role in chronic NASH. Our findings are proved by previous literatures that M2 macrophages increase hepatic injury and fibrosis development in NASH [24].

The upstream mechanism of macrophage TIM3 during the fibrotic stage of NASH is TBP-mediated transcription and β-TrCP-mediated ubiquitination and activation. β-TrCP, a member of the F-box with WD40 repeats subfamilies, specifically recognizes and ubiquitinates proteins that play a key role in cell proliferation, cycle progression, invasion, metastasis, and signal transduction, such as Iκb, β-catenin, Emi1, etc. [25–27]. Previous studies introduced PHAR, a protein-protein interaction inhibitor of NRF2/β-TrCP, which impairs β-TrCP-mediated NRF2 degradation selectively activates NRF2 in the liver, and promotes beneficial effects in NASH. This study suggested that β-TrCP may contribute to the NASH progression [28]. In our study, we found that β-TrCP was high expression in macrophages under fat accumulation situations and promoted membrane TIM3 stability by polyubiquitination. TBP and TBP-associated factors (TAFs), a group of evolutionarily conserved proteins, constitute transcription factor IID (TFIID), which orchestrates the process of multiple proteins involved in transcription initiation [29]. Now, we found that TBP is abnormally activated in macrophages, and directly transcriptionally activates TIM3 at the liver fibrosis stage of NASH.

There are several limitations in this study. First, the MCD model has certain defects because it fails to replicate clinicopathological manifestations such as peripheral insulin resistance and metabolic syndrome. HFD- and western diet (WD)-induced NASH fibrosis models would be better to use to validate MCD model findings. Second, four related proteins have been identified as TIM-3 ligands and interact with the IgV domain of TIM-3 to mediate signal transduction [30]. Here, we did not detect which ligand is involved in MCD-associated NASH fibrosis. Lastly, TBP-mediated-TIM3 transcription and β-TrCP-mediated-TIM3 ubiquitination and activation are obtained in vitro. These findings are not validated in vivo.

In conclusion, our findings demonstrate that macrophage β-TrCP-mediated TIM3 ubiquitination and activation promotes M2 polarization and contribute to chronic fibrosis progression in NAFLD by producing the profibrotic factor TGF-β and supporting the activation of HSCs. Therefore, targeting TIM3 may play an essential role in the immunotherapy of chronic NASH patients with fibrosis.

## Supplementary information

Supplementary Materials and Methods, Supplementary Figures S1-S8 and Table S1-S2

Full and uncropped western blots

## Data Availability

All the data generated during this research were included in the article.
