# Supplementary material for: Macrophage miR-4524a-5p/TBP promotes β-TrCP -TIM3 complex activation and TGFβ release and aggravates NAFLD-associated fibrosis
Source: Cell Death Dis. 2025 Apr 19;16(1):315. doi: 10.1038/s41419-025-07574-4 (PMC12008196; doi:10.1038/s41419-025-07574-4)
Supplement: Supplementary file 1 — Supplementary Materials and Methods, Supplementary Figures S1-S8 and Table S1-S2 [file 41419_2025_7574_MOESM1_ESM.pdf]

# Supplementary Materials

## Macrophage miR-4524a-5p/TBP promotes β-TrCP -TIM3 complex activation and TGFβ release and aggravates NAFLD-associated chronic liver fibrosis

Chunming Li[1,2,3], Lei Fang[1,3], Xingxing Su[1,3], Jie Zhang[1], Haojun Xiong[1], Hongqiang Yu[1], Zhu Zhu[2], Xiaotong Lin[1], Ke Min[1], Di Wu[1], Zhiyu Chen[1*], Jianping Gong[2*], Chuan-Ming Xie[1*#]

# Supplementary Materials and Methods

**HE, Sirius red, immunohistochemical (IHC), and immunofluorescence (IF) staining**

Paraffin-embedded liver tissues from NAFLD patients and modeled complete mice were cut into 3 μm slices. After dewaxing and hydration, tissue sections were used for HE, Sirius red, immunohistochemical (IHC), and immunofluorescence (IF) staining. For HE staining, sections were stained with hematoxylin for 1 min, followed by eosin for 30 s. The sections were then dehydrated and sealed with gradient ethanol. For Sirius red staining, tissue sections were stained with Sirius red solution for 30 min. After that, the stain solution was blotted with absorbent paper, and the slices were sealed with neutral gum. Antigen repair was performed at high temperature and high pressure with sodium citrate solution (Beyotime, China) for IHC staining. After the tissue sections

were naturally cooled to room temperature, endogenous peroxidase was blocked with 3% hydrogen peroxide solution and blocked with goat serum for 30 min at room temperature. Then, the sections were incubated overnight at 4 °C with the following specific primary antibodies: TIM3 (CST, 83382, 1:200), TBP (CST, 44059, 1:200), α-SMA (CST, 19245, 1:1000), F4/80 (CST, 52267, 1:100), CD68 (Proteintech, 66231-2-Ig, 1:1000), and PI3 kinase p85 (Proteintech, 60225-1-Ig, 1:100). Then, a polymer horseradish peroxidase detection system (Zhongshan Goldenbridge Biotechnology) and IF detection system were used for section color development. For the polymer horseradish peroxidase detection system, the sections were incubated with biotin-labeled secondary antibody and streptavidin-labeled horseradish peroxidase at room temperature for 30 min. Then, the signal was visualized with diaminobenzidine, and the nuclei were stained with hematoxylin. The images were observed and photographed using an inverted microscope (Olympus, BX41). For the IF detection system, the slides were incubated with FITC- or PE-labeled secondary antibodies for one hour at room temperature in the dark and sealed with fluorescent sealing tablets containing DAPI (Beyotime, China). Images were observed and collected with a fluorescence microscope (Olympus, BX53F2) in a dark room. Positive cells were counted by ImageJ software.

**Isolation of liver immune cells**

As previously reported, immune cells in the liver were isolated by *in vitro* enzymatic digestionn.[1] Briefly, after the mice were anesthetized with isoflurane, a needle was inserted into the left ventricle, the right atrial appendage was cut open, and normal

saline was perfused at a dose of 1 ml/g to flush blood out of the liver. The gallbladder was then removed, and the liver was cut into 1 $mm^3$ pieces in a 6 cm dish containing normal saline, which was placed in gentle-MACS™ C tubes and digested in 10 ml HBSS containing 0.01% collagenase type IV, 0.02% BSA and 0.001% DNase I for 30 min at 37 °C. The digested mixture was separated with a Gentle-MACS Dissociator (Miltenyi Biotec, Bergisch-Gladbach, Germany). After the termination of digestion with 10 ml of precooled PBS containing 2% FBS, a single-cell suspension was obtained by filtration through a 70 μm cell strainer. Liver nonparenchymal cells were obtained by centrifugation at 50 g for 5 min, followed by removal of the supernatant and centrifugation at 500 × g for 5 min. After washing twice with PBS, the cells were resuspended in 4 ml HBSS, gently overlaid on 33% Percoll solution, and centrifuged at 800 × g for 25 min at 4 °C. Liver immune cells were collected and counted at the bottom of the centrifuge tube.

**Flow cytometry**

Isolated cells were stained with appropriate antibodies for 30 min on ice in the dark. The following antibodies were purchased from BioLegend: APC anti-CD11b (M1/70), AF700 anti-CD45 (30-F11), PE anti-F4/80 (BM8), PE-Cy7 anti-TIM3 (RMT-23). Cell viability was assessed by staining with the Zombie NIR™ Fixable Viability Kit (BioLegend). All flow cytometry assays were performed in FACS buffer containing PBS with 2 mM EDTA and 2% FBS on a BD LSRFortessa flow cytometer (BD Biosciences) and analyzed with FlowJo software (Tree Star).

**Isolation of Kupffer cells**

Primary Kupffer cells (KCs) of the liver were isolated following the method of Li et al.[2] with minor modifications. After the mice were anesthetized, laparotomy was performed on an ultraclean table to fully expose the portal vein and inferior vena cava. After inserting the needle through the portal vein, the liver was perfused with EGTA solution, and liver filling indicated that the needle was successfully cannulated. The inferior vena cava was cut open. After that, 25 ml of streptomycin protease solution (0.4 mg/ml) and 35 ml of collagenase IV solution (0.4 mg/ml) were infused sequentially. After in situ perfusion, livers were removed and sheared into a sterile petri dish containing 5 ml of streptomycin protease (0.4 mg/ml), collagenase IV (0.4 mg/ml), and 0.001% DNase I. After 20 min of *in vitro* digestion, digestion was terminated by passing the digested solution through a 70 μm cell filter and adding 10 ml of Gey's balanced salt solution (GBSS). The supernatant was removed after centrifugation at 500 × g for 5 min at 4 °C, and the precipitate was resuspended in 10 ml GBSS and centrifuged at 50 g for 5 min at 4 °C to remove liver parenchymal cells. The supernatant was centrifuged at 500 × g for 5 min at 4 °C and then washed twice with GBSS. The supernatant was then seeded at $1\times10^7$/well in a mixture containing 10% fetal bovine serum (FBS, Gibco, USA) and 100 units/ml penicillin and streptomycin (PS, HyClone, USA) in Dulbecco's modified Eagle's medium (DMEM, Gibco, USA) and incubated for 2 h in a humidified incubator with 5% CO2 at 37 °C. Nonadherent cells were removed by light washing with PBS, and adherent cells were KCs.

**Cell culture and transfection**

The mouse macrophage cell line RAW264.7 and mouse HSC line JS1 were obtained from the American Type Culture Collection (ATCC). Short Tandem Repeat profiling was conducted on all cell lines. All cell lines and primary KCs were cultured in DMEM supplemented with 10% FBS and 100 units/ml PS in an incubator with 5% $CO_2$ at 37 °C. Cells were transfected with TBP plasmids, siRNA targeting Havcr2 (siHavcr2), siRNA targeting TBP (siTBP), miR-4524a-5p mimics or the associated control by Lipofectamine 2000 (Invitrogen). The mouse TBP coding sequences were cloned into the pcDNA3.1 vector (GeneChem, Shanghai, China). siRNAs were obtained from GenePharma (Shanghai, China). The sequences of siRNAs were as follows: Havcr2 siRNA-1 (5' -CCAGCAGAUACCAGCUAAATT- 3'), Havcr2 siRNA-2 (5' - CCCUGGUCUUAUGAAUGAUAA- 3'), TBP siRNA-1 (5' - CCAGAAUUAUUUCCUGGAUUA- 3'), TBP siRNA-2 (5' - CAGCCUCAGUACAGCAAUCAA- 3'), and miR-4524a-5p mimics (5' - CCAGCAGAUACCAGCUAAATT- 3').

**Quantitative reverse transcriptase polymerase chain reaction (qRT–PCR)**

Total RNA was extracted from cultured cells and liver tissues using TRIzol (TaKaRa). Subsequently, the PrimeScript RT Reagent Kit (TaKaRa) was used to remove genomic DNA and reverse it into cDNA. TB Green Premix Ex Taq II (TaKaRa) was used to detect mRNA expression levels in tissues and cells. The PCR cycle programs were as follows: 95 °C for 30 s and 40 cycles at 95 °C for 5 s and 60 °C for 1 min. A miRNA reverse transcription kit (GenePharma E01006, Shanghai, China) was used for reverse transcription and qRT–PCR detection of miRNA. β-Actin was used as the internal

control for mRNA, while U6 was used as the internal reference for miRNA. The primer sequences used in this experiment are shown in **Supplementary Table 2.**

**Western blot**

The total proteins of liver tissues or cells were extracted using RIPA lysis buffer (Beyotime, China). Briefly, liver tissues or cells were lysed on ice for 15 min in RIPA lysis buffer and then centrifuged at 12,000 g at 4 °C for 10 min. The protein concentration was determined using a bicinchoninic acid kit (Beyotime, China) according to the manufacturer's instructions. Proteins were denatured using loading buffer containing SDS (Beyotime, China) and boiled at 95 °C for 15 min. The proteins were separated by sodium dodecyl sulfate–polyacrylamide gel electrophoresis (SDS–PAGE) and transferred to a nitrocellulose transfer membrane (GE Healthcare). The membranes were blocked with Tris-buffered saline Tween-20 (TBST) solution containing 5% nonfat milk for 1 h and were incubated overnight at 4 °C with primary antibodies. After washing with TBST, the membranes were incubated with the secondary antibody horseradish peroxidase-conjugated anti-rabbit immunoglobulin G (IgG) (CST, 7074, 1:5000) or anti-mouse immunoglobulin G (IgG) (CST, 7076, 1:5000) at room temperature for 1 h. After washing again, the protein bands were visualized using a Bio-Rad Image Analysis System (Bio-Rad, USA) with an enhanced chemiluminescence fluorescence detection kit (GE HealthCare, USA). The primary antibodies were as follows: TIM3 (CST, 83382, 1:1000), TBP (CST, 44059, 1:1000), phospho-Akt (CST, 4060, 1:1000), Akt (CST, 4691, 1:1000), phospho-mTOR (CST, 5536, 1:1000), mTOR (CST, 2983, 1:1000), phospho-p70 S6k (CST, 9234, 1:1000),

p70 S6k (CST, 9202, 1:1000), phospho-4E-BP1 (CST, 2855, 1:1000), 4E-BP1 (CST, 9644, 1:1000), PI3 kinase p85 (CST, 4292, 1:1000), α-SMA (CST, 19245, 1:1000), PDGFRβ (CST, 3160, 1:1000), and β-actin (Proteintech, 66008-1-Ig, 1:5000).

**Coimmunoprecipitation (Co-IP) assay**

RAW264.7 cells were lysed in lysis buffer (20 mM Tris-HCl, pH 8.0, 100 mM NaCl, and 1% NP-40) for western blotting and IP (Beyotime, China) on ice for 20 min, and cell debris was removed after centrifugation at 12,000 × g for 10 min at 4 °C. Five micrograms of primary antibodies and 40 μl of Protein A/G PLUS-Agarose (Santa Cruz, CA) were added to the cell lysates, and they were incubated with rotation at 4 °C overnight. The beads were washed four times with lysis buffer, loading buffer containing SDS (Beyotime, China) was added, and then the beads were boiled at 100 °C for 10 min. Denatured proteins were analyzed by Western blotting.

**In vivo ubiquitination assay**

HEK293T cells were transfected with His-Ub, Flag-β-TrCP, and HA-TIM3 plasmids to examine the ubiquitination of TIM3 by β-TrCP. After 72 h of transfection, the cells were treated with 10 mM MG132 for 4 h and harvested for the in vivo ubiquitination assay. The cells were lysed in buffer A (6 mol/L guanidinium-HCl, 0.1 mol/L Na2HPO4/NaH2PO4, 10 mmol/L Tris-HCl (pH8.0), 5 mmol/L imidazole and 10 mmol/L β-mercaptoethanol) and incubated with 50 mL of Ni-NTA beads (Qiagen, Valencia, CA) overnight. The beads were washed with buffer A, buffer B (8 mM urea, 0.1 M Na2HPO4/NaH2PO4, 10 mM Tris/HCl (pH 8.0), and 10 mM β-mercaptoethanol), buffer C (8 mM urea, 0.1 M Na2HPO4/NaH2PO4, 10 mM Tris/HCl

(pH 6.3), 10 mM β-mercaptoethanol) containing 0.2% Triton X-100, and buffer C containing 0.1% Triton X-100. Then the ubiquitinated proteins were eluted with buffer D (200 mM imidazole, 0.15 M Tris-HCl (pH 6.7), 30% glycerol, 0.72 M β-mercaptoethanol, and 5% SDS), boiled at 95 °C for 4 min, and analyzed by Western blotting.

**Cell proliferation assay**

The cell-counting kit-8 assay (CCK-8, Bimake, USA) was used to analyze cell proliferation. Briefly, JS1 cells were seeded at 3000 cells/well in 96-well plates. After treatment for 0, 24, 48, and 72 h, cells were exposed to 10 μl/well CCK-8 reagent and incubated at 37 °C for 2 h. Then, the absorbance at 450 nm was detected using an enzyme calibration system (Thermo Fisher Scientific, USA).

**Chromatin immunoprecipitation (ChIP)**

ChIP experiments were performed using a ChIP Assay Kit (Beyotime, China) according to the manufacturer's instructions. RAW264.7 cells were cross-linked with 1% formaldehyde at 37 °C for 10 min, and the reaction was terminated by adding glycine solution buffer. After washing with PBS, cells were scraped off and lysed fully with SDS lysis buffer. The cell chromatin was then broken into 200-500 bp fragments using an ultrasonic splitter. Subsequently, sonically treated samples were subjected to ChIP containing Protein A+G Agarose/Salmon Sperm DNA and 5 μg rabbit anti-TBP (CST, 44059) or rabbit IgG control (CST, 3900) antibody. The samples were incubated in dilution buffer overnight at 4 °C. DNA fragments were uncross-linked by incubation in ChIP elution buffer containing 5 M NaCl for 4 h at 65 °C and subsequent purifications

of DNA were performed with DNA extraction phenol reagent for detection by PCR. Primer sequences for ChIP–qPCR are shown in **Supplementary Table 2**.

**Luciferase reporter assay**

The 3'-untranslated region (UTR) or the mutant sequence of human TBP was cloned into the pmirGLO vector. RAW264.7 cells were seeded in 24-well plates and transfected with miR-4524a-5p mimics, wild-type TBP 3'-UTR, or mutant TBP 3'-UTR luciferase reporter plasmids. After 48 h, luciferase was measured using a Dual-Luciferase Assay Kit (Promega) according to the manufacturer's instructions.

**Statistical analysis**

GraphPad Prism Version 8.0 (CA, USA) and SPSS 25 Software (SPSS Inc., New York, USA) were used to analyze the trial data. The measurement data are expressed as the mean ± SEM. The Shapiro-Wilk method was used to check if the data conformed to a normal distribution, and the F-test was performed to estimate the variation within each group of data. All the data were normally distributed. The variance that are being statistically compared was similar between the groups expect Figure 7C-H. The unpaired t test was performed for comparisons between two groups, while analysis of variance (ANOVA) with Tukey's or Bonferroni's multiple comparisons test was used for comparisons among multiple groups. Logistic regression was used to analyze the correlation between different factors. The Kaplan–Meier method was used for the survival analysis. $P < 0.05$ indicated statistical significance.

# Supplementary Figures

## Figure S1

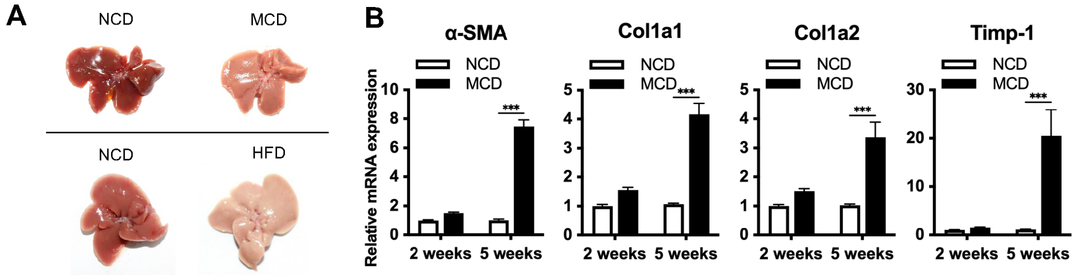


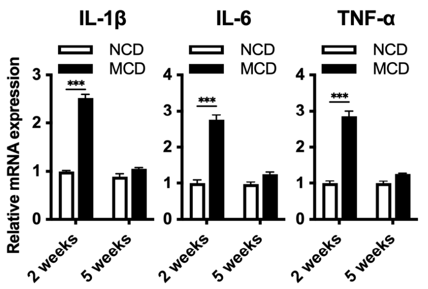


**D**

| Score | NCD | MCD | NCD | HFD |
|---|---|---|---|---|
| NAS | 0.63±0.52 | 6±0.76 | 0.5±0.55 | 6±0.76 |
| Steatosis | 0 | 3 | 0 | 3 |
| Ballooning | 0 | 1.25±0.46 | 0 | 1.33±0.52 |
| Inflammation | 0.63±0.52 | 1.75±0.46 | 0.5±0.55 | 2 |
| Fibrosis | 0 | 2.88±0.64 | 0 | 2.67±0.52 |

NAS, NAFLD Activity Score.

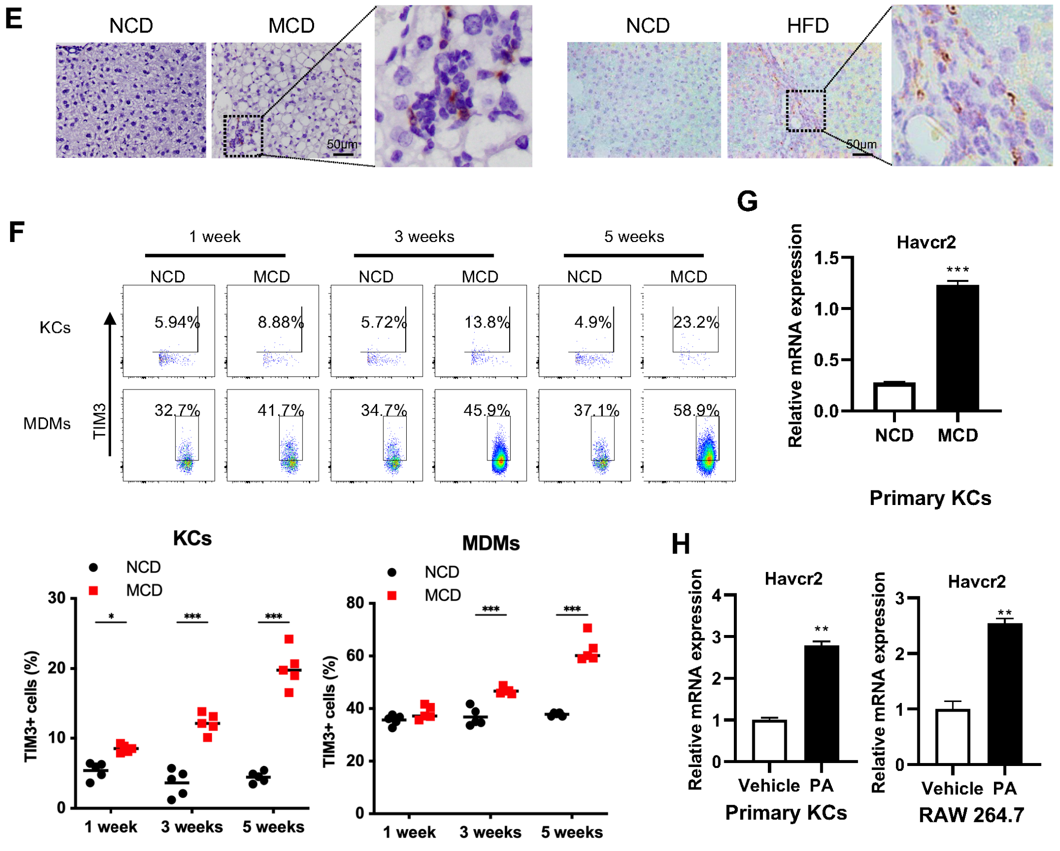

**Figure S1. TIM3 was highly expressed in liver macrophages in NASH fibrosis mice**

(A) Representative gross liver images of mice fed a negative control diet (NCD) and methionine and choline-deficient (MCD) diet for 5 weeks, and NCD and high-fat diet (HFD) for 26 weeks (n=6/group). **(B)** mRNA expression levels of α-SMA, Col1a1, Col1a2, PDGF-β, and Timp of mice fed with NCD or MCD for 2 or 5 weeks were determined by qPCR (n=4/group). **(C)** mRNA expression levels of IL-1β, IL-6, and TNF-α were determined by qPCR in mice fed with NCD or MCD for 2 or 5 weeks (n=4/group). **(D)** the NAFLD activity score (NAS) and fibrosis score of liver tissues in (A) (n=6/group). **(E)** Representative IHC-stained sections showed increased TIM3 expression levels in the MCD/HFD group mice compared with the NCD group mice. **(F)** Isolated hepatic F4/80$^{high}$CD11b$^{low}$ Kupffer cells (KCs) and F4/80$^{low}$CD11b$^{high}$ monocyte-derived macrophages (MDMs) from 1, 3, and 5-week NCD/ MCD-fed mice were analyzed for TIM3 expression by flow cytometry. Quantification of the percentage of TIM3-positive cells (n=5/group). **(G)** qPCR was used to measure the mRNA expression levels of Havcr2 in isolated primary KCs from mice treated as described in (A) (n=3/group). **(H)** Havcr2 mRNA expression levels in primary KCs isolated from NCD-fed WT mice and RAW264.7 cells treated with 0.5 mM palmitic acid (PA) for 24 h. Data are presented as the mean ± SEM; significance determined by Student's unpaired t test (G, H) and two-way ANOVA with Bonferroni's multiple comparisons test (B, C, F). *p <0.05, **p <0.01, ***p <0.001.

## Figure S2

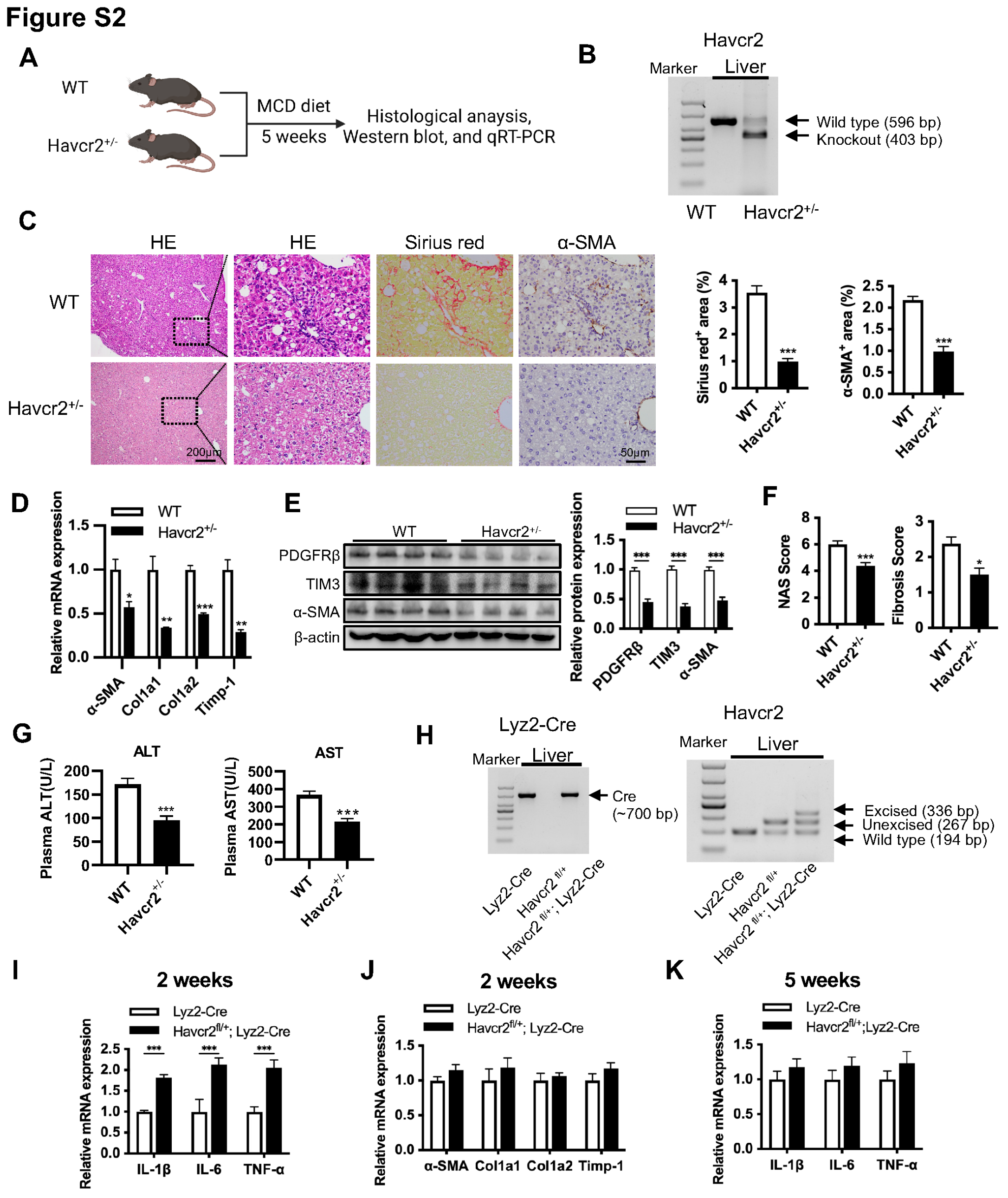


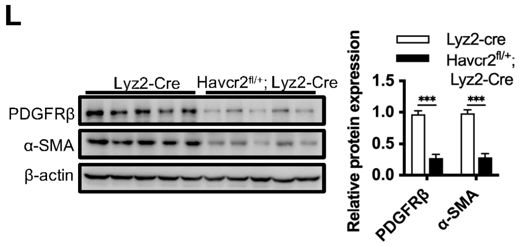


**M**

| Score | Lyz2-Cre | Havcr2$^{fl/+}$; Lyz2-Cre | WT | Havcr2$^{+/-}$ |
|---|---|---|---|---|
| NAS | 5.57±0.79 | 3.57±0.79 | 5.25±0.46 | 4.38±0.74 |
| Steatosis | 2.71±0.49 | 1.71±0.49 | 2.63±0.52 | 2.50±0.54 |
| Ballooning | 1.43±0.54 | 1.14±0.38 | 1.13±0.35 | 0.75±0.46 |
| Inflammation | 1.43±0.54 | 0.71±0.49 | 1.50±0.54 | 1.13±0.35 |
| Fibrosis | 2.43±0.54 | 1.14±0.38 | 2.38±0.52 | 1.50±0.54 |

NAS, NAFLD Activity Score.

**Figure S2. TIM3 (haplo)insufficiency protects against NASH-induced liver damage and fibrosis.**

**(A-G)** Havcr2 knockdown (Havcr2$^{+/-}$) mice and WT mice were fed an MCD diet for 5 weeks (n=8/group). DNA was isolated from the liver tissue of the indicated genotype and subjected to PCR genotyping for Havcr2$^{+/-}$ (B). IHC staining and Sirius red staining showing fibrosis levels (C). The mRNA expression levels of α-SMA, Col1a1, Col1a2, and Timp-1 were determined by qPCR (D). The protein expression levels of TIM3, PDGFRβ, and α-SMA in liver tissues were detected by western blot (E). The relative protein expression was normalized to the level of β-actin (E). Graphs show NAFLD activity score (NAS), fibrosis score, serum ALT, and serum AST levels (F, G). **(H)** DNA was isolated from the liver tissue of the indicated genotype and subjected to PCR genotyping for the Lyz2-Cre (left) and Havcr2$^{fl/+}$; Lyz2-Cre (right). **(I)** mRNA expression levels of IL-1β, IL-6, and TNF-α were determined by qPCR in 2-week MCD-fed Lyz2-Cre and Havcr2$^{fl/+}$; Lyz2-Cre mice (n=4/group). **(J)** mRNA expression levels of α-SMA, Col1a1, Col1a2, and Timp-1 were determined by qPCR of mice in (I). **(K)** mRNA expression levels of IL-1β, IL-6, and TNF-α were determined by qPCR in 5-week MCD-fed Lyz2-Cre and Havcr2$^{fl/+}$; Lyz2-Cre mice (n=4/group). **(L)** The protein expression levels of PDGFRβ and α-SMA in liver tissues from Lyz2-Cre and Havcr2$^{fl/+}$; Lyz2-Cre mice were detected by western blot. The relative protein expression was normalized to the level of β-actin. **(M)** The NAS and fibrosis score of liver tissues of indicated mice were quantified. Data are presented as the mean ± SEM; significance determined by Student's unpaired t test (C, F, G) and two-way ANOVA

with Bonferroni's multiple comparisons test (D, E, I-L). *p $<0.05$, **p $<0.01$, ***p $<0.001$.

## Figure S3

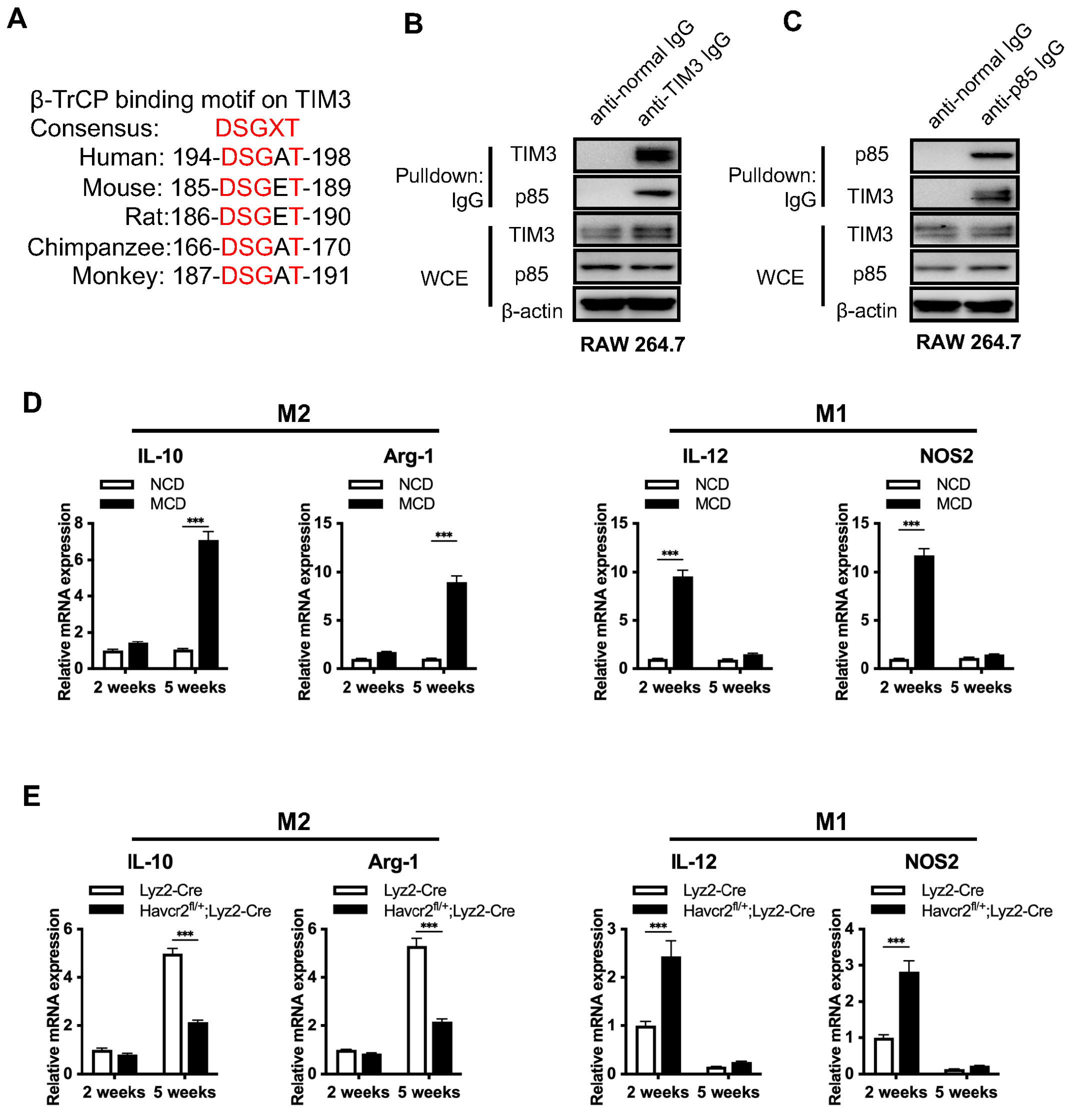


**Figure S3. TIM3 has β-TrCP consensus binding motif, and TIM3 binds with PI3K to induce M2 polarization**

**(A)** TIM3 has an evolutionarily conserved consensus binding motif (DSGXT) for β-TrCP. **(B, C)** TIM3 and p85 interaction was analyzed by Co-IP in RAW264.7 cells. **(D)** mRNA expression levels of IL-10, Arg-1, IL-12, and NOS2 of mice fed with NCD or MCD for 2 or 5 weeks were determined by qPCR (n=4/group). **(E)** mRNA expression

levels of IL-10, Arg-1, IL-12, and NOS2 were determined by qPCR in MCD-fed Lyz2-Cre and Havcr2$^{fl/+}$; Lyz2-Cre for 2 or 5 weeks (n=4/group). Data are presented as the mean ± SEM; significance determined by two-way ANOVA with Bonferroni's multiple comparisons test (D, E). ***p <0.001.

## Figure S4

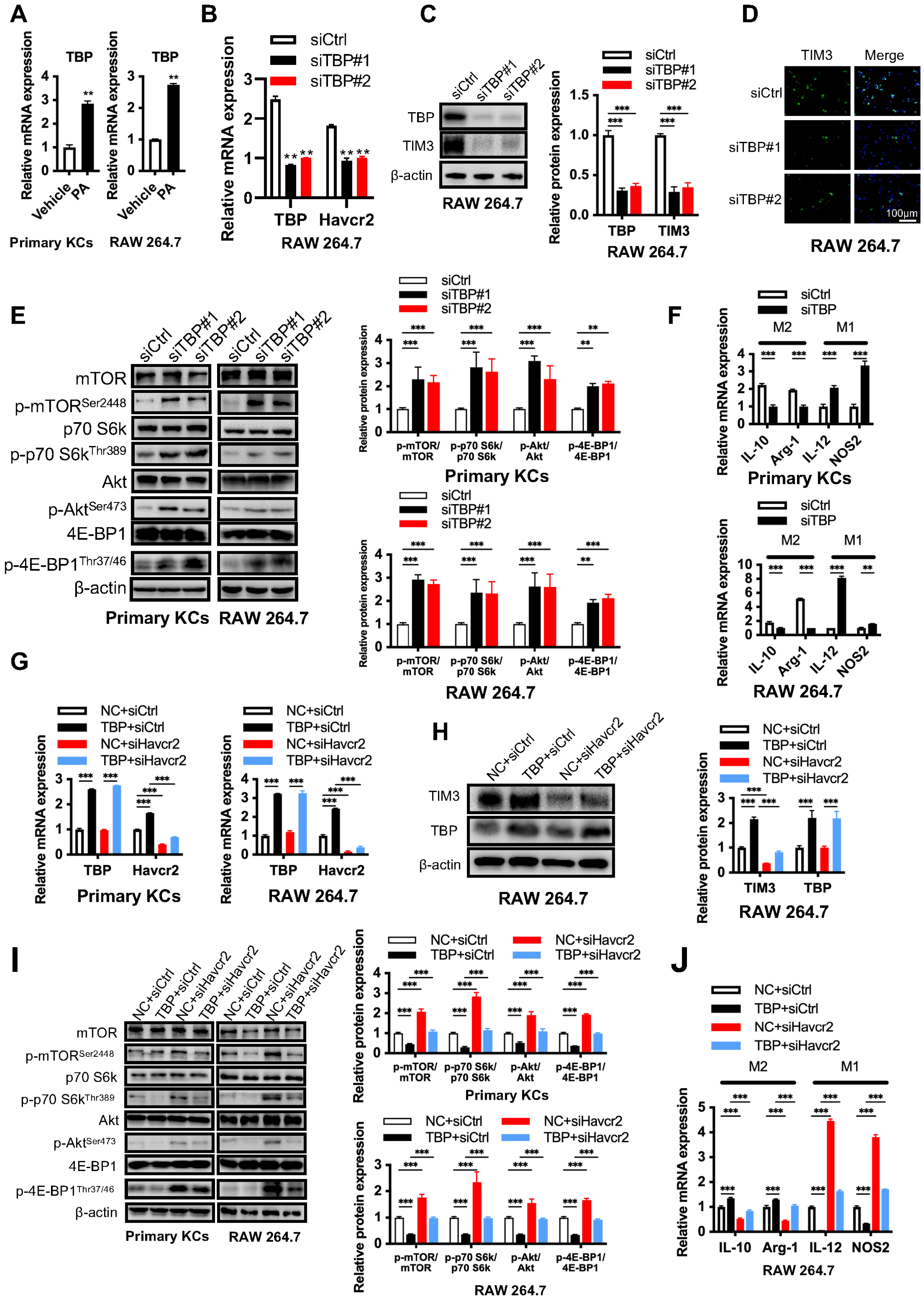


255

**Figure S4. Elevated TIM3 is attributed to the TBP expression in macrophages**

**(A)** TBP mRNA expression levels in primary NCD-KCs and RAW264.7 cells after exposure to 0.5 mM PA for 24 h. **(B-F)** RAW264.7 cells were transfected with a non-targeting siRNA Control (siCtrl) or siRNA targeting TBP (siTBP) for 48 h. The expression levels of TIM3, TBP and AKT/mTOR were examined by qPCR (B) or western blot (C, E). The relative protein expression was normalized to the level of β-actin (C, E). Representative immunocytochemical image showed localization of TIM3 (D). Macrophage polarization markers at mRNA level was analyzed by qPCR (F). **(G-J)** RAW264.7 cells were transfected with TBP plasmid or siHavcr2 for 48 h. Havcr2 and TBP at mRNA (G) or protein levels (H) were detected by qPCR or western blot. AKT/mTOR pathway was analyzed by western blot (I). The relative protein expression was normalized to the level of β-actin (H, I). Macrophage polarization markers at mRNA level were detected by qPCR (J) (n=3/group). Data are presented as mean ± SEM; significance determined by Student's unpaired t-test (A) and Two-way ANOVA with Bonferroni's multiple comparisons test (B, C, E-J). $^{**}p<0.01$, $^{***}p<0.001$.

**Figure S5**

**A**

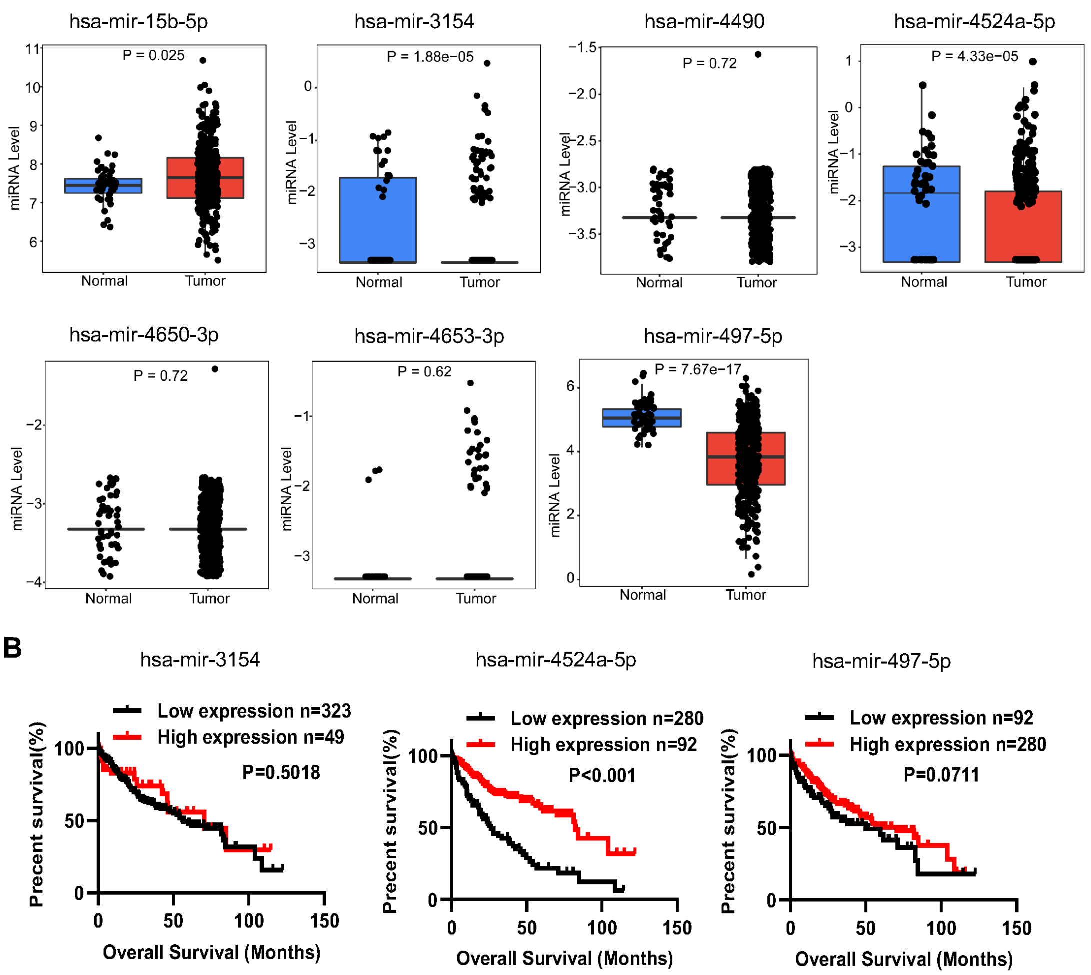


**Figure S5. Differential expression and survival analysis of the miRNA that might interact with TBP**

**(A)** Relative expression of 7 possible miRNAs (miR-15b-5p, miR-3154, miR-4490, miR-4524a-5p, miR-4650-3p, miR-4653-3p, and miR-497-5p) that bind to TBP in tumor (n=372) and normal (n=50) tissues of HCC patients from TCGA database. **(B)** Kaplan-Meier analysis of overall survival of miR-3154, miR-4524a-5p and miR-497-5p expression in HCC patients.

## Figure S6

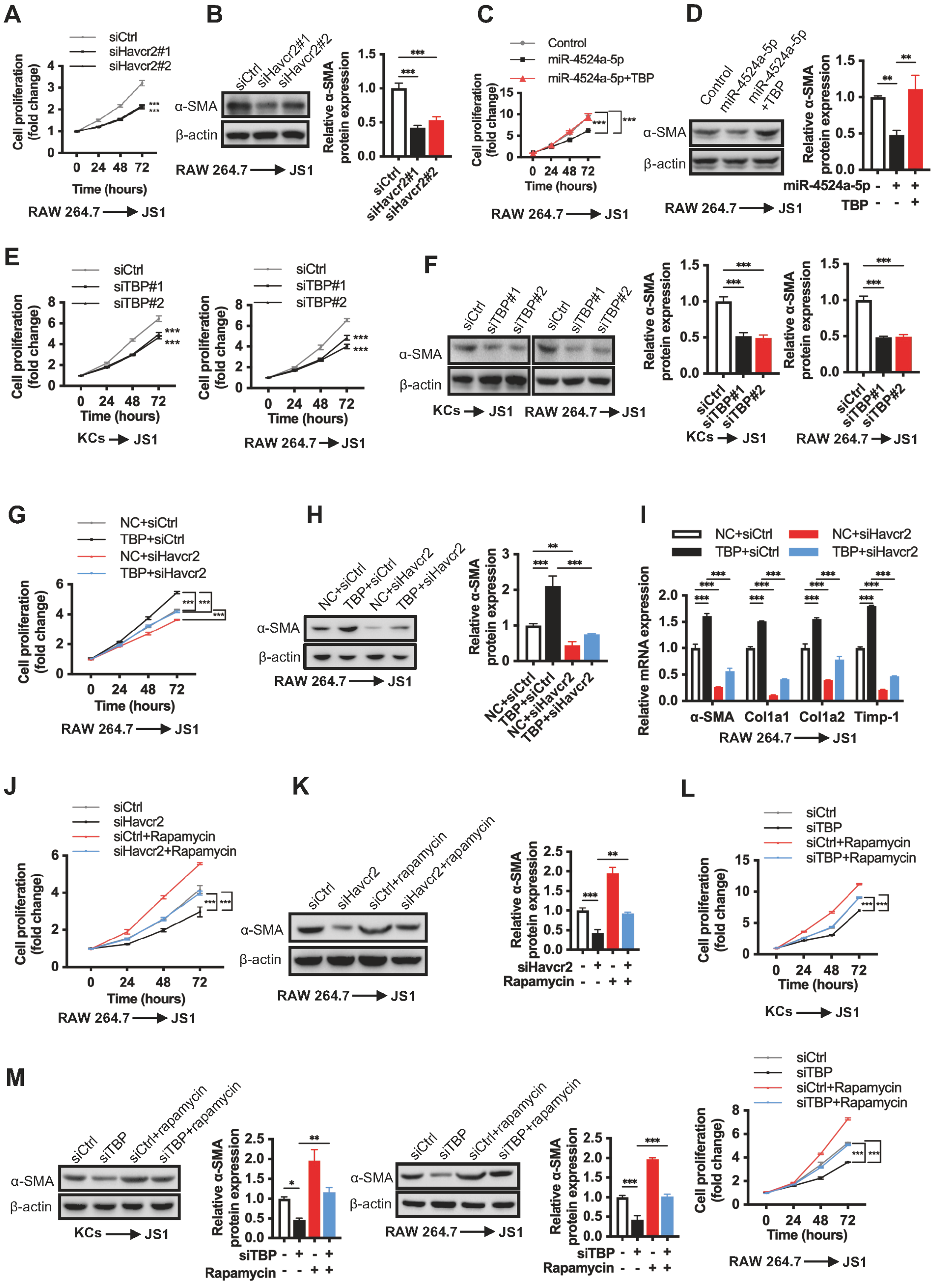


279

**Figure S6. Macrophage miR-4524a-5p/TBP/TIM3/mTOR signaling pathway mediates HSCs proliferation and activation**

**(A, B)** RAW264.7 cells were treated with siCtrl or siHavcr2 for 48 h, and the Conditioned medium (CM) were harvested and added to mouse JS1 cells. JS1 cells were continued in the medium for the indicated time intervals, and cell proliferation was assessed by CCK-8 (A), α-SMA in whole cell lysates was determined by western blot (B) (n=3/group). The relative protein expression was normalized to the level of β-actin (B). **(C, D)** RAW264.7 cells were transfected miR-4524a-5p with or without TBP overexpression plasmid for 48 h, and the CM was harvested and added to mouse JS1 cells for different time intervals. JS1 cells proliferation was assessed by CCK-8 (C). α-SMA in whole cell lysates was determined by western blot (D) (n=3/group). The relative protein expression was normalized to the level of β-actin (D). **(E, F)** Primary KCs and RAW264.7 cells were transfected with siCtrl or siTBP for 48 h, and the CM was collected and added to JS1 cells. JS1 cells were continued in the medium at specified time points, and cell proliferation was assessed by CCK-8 (E), α-SMA in whole cell lysates was determined by western blot (F) (n=3/group). The relative protein expression was normalized to the level of β-actin (F). **(G-I)** RAW264.7 cells were transfected with the TBP overexpression plasmid and siHavcr2 for 48 h. The CM was collected and added to JS1 cells. Cell proliferation was assessed by CCK-8 (G). The protein expression level of α-SMA in JS1 was determined by western blot (H), and the mRNA expression levels of α-SMA, Col1a1, Col1a2, and Timp in JS1 cells were measured by qPCR (I) (n=3/group). The relative protein expression was normalized to

the level of β-actin (H). **(J-M)** Primary KCs isolated from MCD-fed WT mice and RAW264.7 cells were transfected siHavcr2/siTBP with or without mTOR inhibitor rapamycin (100 nM) for 48 h, and the CM was collected and added to JS1 cells. Cell proliferation was assessed by CCK-8 (J, L). The α-SMA in whole cell lysates was determined by western blot (K, M) (n=3/group). The relative protein expression was normalized to the level of β-actin (K, M). Data are presented as mean ± SEM; One-way ANOVA with Bonferroni's multiple comparisons test was used in B, D, F, H, K, M; Two-way ANOVA with Bonferroni's multiple comparisons test was used in A, C, E, G, I, J, L. *p<0.05, **p<0.01, ***p<0.001.

Figure S7

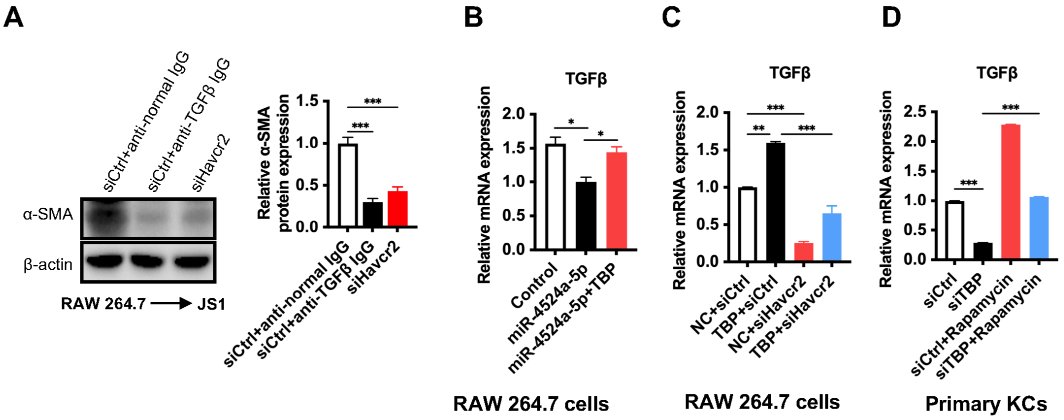


**Figure S7. Macrophage miR-4524a-5p/TBP/TIM3/mTOR signaling pathway mediates TGF-β production**

**(A)** RAW264.7 cells were treated with anti-TGF-β antibody or siHavcr2 for 48 h, and the CM was collected and added to JS1 cells. The α-SMA in whole cell lysates was determined by western blot (n=3/group). The relative protein expression was normalized to the level of β-actin. **(B)** RAW264.7 were transfected miR-4524a-5p with or without TBP overexpression plasmid for 48 h, and qPCR was used to measure TGF-β expression levels (n=3/group). **(C)** RAW264.7 were transfected with the TBP overexpression plasmid and siHavcr2 for 48 h, and qPCR was used to measure TGF-β expression levels (n=3/group). **(D)** Primary KCs were transfected siTBP with or without mTOR inhibitor rapamycin (100 nM) for 48 h, and qPCR was used to measure TGF-β expression levels (n=3/group). Data are presented as mean ± SEM; One-way ANOVA with Bonferroni's multiple comparisons test was used in A-D. *p<0.05, **p<0.01, ***p<0.001.

## Figure S8

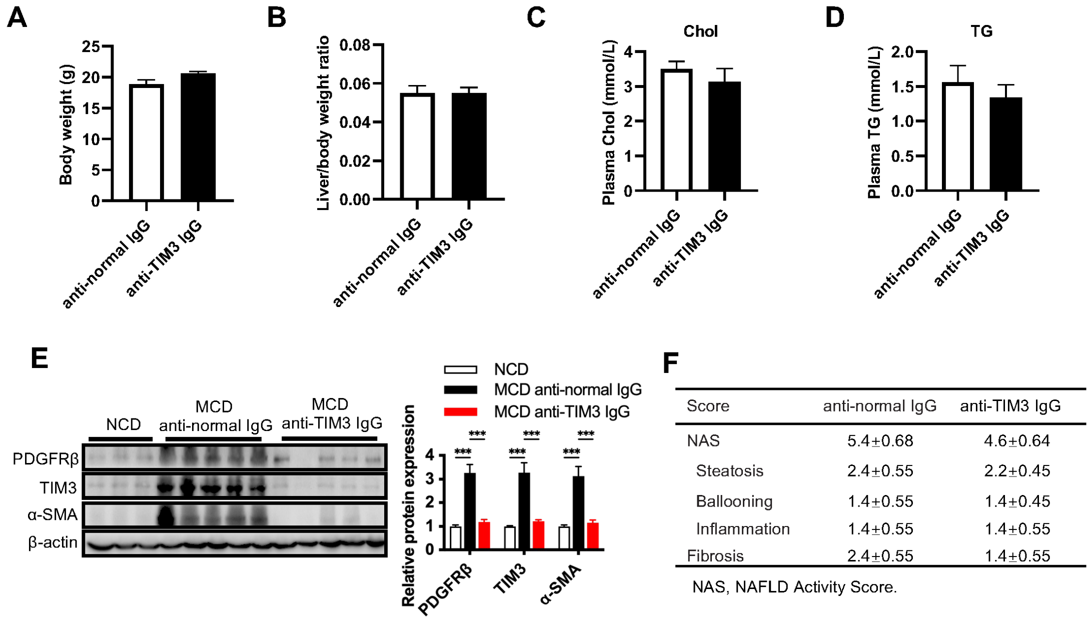


| Score | anti-normal IgG | anti-TIM3 IgG |
|---|---|---|
| NAS | 5.4±0.68 | 4.6±0.64 |
| Steatosis | 2.4±0.55 | 2.2±0.45 |
| Ballooning | 1.4±0.55 | 1.4±0.45 |
| Inflammation | 1.4±0.55 | 1.4±0.55 |
| Fibrosis | 2.4±0.55 | 1.4±0.55 |

NAS, NAFLD Activity Score.

**Figure S8. Blockade of TIM3 signaling suppresses fibrosis but did not affect liver fat metabolism during NASH development**

**(A-F)** C57BL/6 mice fed an MCD diet that were intraperitoneally injected with anti-TIM3 IgG or normal IgG once every five days. Graphs show body weight, liver/body weight ratio, and plasma Chol and TG levels of mice (A-D). The protein expression levels of TIM3, PDGFRβ, and α-SMA in liver tissues of each group were detected by western blot (E). The relative protein expression was normalized to the level of β-actin (E). The NAS and fibrosis score of mice (F). Data are presented as mean ± SEM; Student's unpaired t-test was used in A-D; Two-way ANOVA with Bonferroni's multiple comparisons test was used in E. ***$p<0.001$.

# Supplementary Tables

**Supplementary Table 1. Clinical and pathological characteristics of patients with NAFLD/NASH**

| Characteristics | All (n=48) | No advanced fibrosis (stage 0-2) (n=22) | Advanced fibrosis (stage 3-4) (n=26) |
|---|---|---|---|
| **Age (years)** | 60.27±10.26 | 60.95±9.48 | 59.69±11.02 |
| **Gender, n** | | | |
| Female | 11 | 7 | 4 |
| Male | 37 | 15 | 22 |
| **Laboratory tests** | | | |
| ALT (U/L) | 51.93±69.03 | 23.07±9.36 | 76.35±86.79 |
| AST (U/L) | 42.88±40.35 | 22.45±7.39 | 60.15±48.35 |
| GGT (U/L) | 69.47±73.89 | 38.66±19.66 | 95.54±91.64 |
| Total bilirubin (mg/ml) | 14.16±5.33 | 11.65±3.91 | 16.28±5.52 |
| Albumin (g/dl) | 39.98±5.82 | 41.61±5.7 | 38.6±5.67 |
| **Histology** | | | |
| **NAS, n** | | | |
| Steatosis | | | |
| 0 | 1 | 1 | 0 |
| 1 | 25 | 13 | 12 |

| | | | |
|---|---|---|---|
| 2 | 17 | 6 | 11 |
| 3 | 5 | 2 | 3 |
| Ballooning | | | |
| 0 | 4 | 3 | 1 |
| 1 | 28 | 11 | 17 |
| 2 | 16 | 8 | 8 |
| Inflammation | | | |
| 0 | 1 | 1 | 0 |
| 1 | 26 | 16 | 10 |
| 2 | 16 | 5 | 11 |
| 3 | 5 | 0 | 5 |
| Fibrosis, n | | | |
| 0 | 4 | 4 | 0 |
| 1 | 10 | 10 | 0 |
| 2 | 8 | 8 | 0 |
| 3 | 14 | 0 | 14 |
| 4 | 12 | 0 | 12 |

ALT, alanine aminotransferase; AST, aspartate aminotransferase; GGT, gamma-glutamyltransferase; NAFLD, non-alcoholic fatty liver disease; NAS, NAFLD Activity Score; NASH, non-alcoholic steatohepatitis.

**Supplementary Table 2. The Primer sequences for qRT-PCR**

| Primer name | Prime direction | Primer sequence (5 '- 3') |
|---|---|---|
| **α-SMA (mouse)** | Forward | CCGCCATGTATGTGGCTATT |
| | Reverse | CAGTTGTACGTCCAGAGGCATA |
| **Col1a1 (mouse)** | Forward | TAAGGGTCCCCAATGGTGAGA |
| | Reverse | GGGTCCCTCGACTCCTACAT |
| **Col1a2 (mouse)** | Forward | CCAGCGAAGAACTCATACAGC |
| | Reverse | GGACACCCCTTCTACGTTGT |
| **PDGFRβ (mouse)** | Forward | TCCCACATTCCTTGCCCTTC |
| | Reverse | GCACAGGGTCCACGTAGATG |
| **Timp (mouse)** | Forward | CGAGACCACCTTATACCAGCG |
| | Reverse | GGCGTACCGGATATCTGCG |
| **PD-1 (mouse)** | Forward | TCCAACTGGTCGGAGGATCT |
| | Reverse | TGTATGATCTGGAAGCGGGC |
| **PD-L1 (mouse)** | Forward | ACAGCAACTTCAGGGGGAGA |
| | Reverse | CGGTATGGGGCATTGACTTT |
| **Havcr2 (mouse)** | Forward | GGAGTCTCTGCTGGGTTGAC |
| | Reverse | TCAGAGCGAATCCTGACTGC |
| **CTLA4 (mouse)** | Forward | AGAACCATGCCCGGATTCTG |
| | Reverse | CATCTTGCTCAAAGAAACAGCAG |
| **LAG3 (mouse)** | Forward | CTGCTTTGGGAAGCTCCAGT |

| | | |
|---|---|---|
| | Reverse | CCAGGTTGGGGGATTTGAGG |
| **TIGIT (mouse)** | Forward | GCAGGCACGATAGATACAAAG |
| | Reverse | TCACTGAAGACTGAAGCGACA |
| **IL-10 (mouse)** | Forward | CTTACTGACTGGCATGAGGATCA |
| | Reverse | GCAGCTCTAGGAGCATGTGG |
| **Arg-1 (mouse)** | Forward | GGAATCTGCATGGGCAACCTGTGT |
| | Reverse | AGGGTCTACGTCTCGCAAGCCA |
| **IL-12 (mouse)** | Forward | CTGTGCCTTGGTAGCATCTATG |
| | Reverse | GCAGAGTCTCGCCATTATGATTC |
| **NOS2 (mouse)** | Forward | GTTCTCAGCCCAACAATACAAGA |
| | Reverse | GTGGACGGGTCGATGTCAC |
| **IL-1β (mouse)** | Forward | GCAACTGTTCCTGAACTCAACT |
| | Reverse | ATCTTTTGGGGTCCGTCAACT |
| **IL-6 (mouse)** | Forward | CCACTTCACAAGTCGGAGGCTTA |
| | Reverse | GCAAGTGCATCATCGTTGTTCATAC |
| **TNF-α (mouse)** | Forward | ATTCGAGTGACAAGCCTGTAGCCCA |
| | Reverse | CTGGGAGTAGACAAGGTACAACCCA |
| **YY1 (mouse)** | Forward | CTCCTGCAGCCCTGGGCGCATC |
| | Reverse | GGTAAGCCCTTTAGCGCCTC |
| **SP1 (mouse)** | Forward | AGAACCCACAAGCCCAGACAATC |
| | Reverse | CTCCTTCTCCACCTGCTGTCTCA |
| **ELF1 (mouse)** | Forward | CAAGTAACGGCATGGAGGAC |

| | | |
|---|---|---|
| | Reverse | CTGTAAGGGTGATGTCGTC |
| **TBP (mouse)** | Forward | TGCACAGGAGCCAAGAGTGAA |
| | Reverse | CACATCACAGCTCCCCACCA |
| **TGF-β(mouse)** | Forward | CTCCCGTGGCTTCTAGTGC |
| | Reverse | GCCTTAGTTTGGACAGGATCTG |
| **PDGFβ(mouse)** | Forward | GGTGAGCAAGGTTGTAATGG |
| | Reverse | GGAGGCAATGGACAGACAA |
| **Ccl2 (mouse)** | Forward | TTAAAAACCTGGATCGGAACCAA |
| | Reverse | GCATTAGCTTCAGATTTACGGGT |
| **Havcr2-1 (mouse)** | Forward | GGGCACCGTGGGACATATAT |
| | Reverse | AGGCTGGCCTCAAACTTCTC |
| **Havcr2-2 (mouse)** | Forward | CCCCACACACACACACTTCA |
| | Reverse | ACAAGTCCCCTGAGCCCTAG |
| **Havcr2-3 (mouse)** | Forward | AGATGTCTTAGCAGGGTTTCCC |
| | Reverse | CAGTGCCCAGGCTGTGTAAT |
| **Havcr2-4 (mouse)** | Forward | TCGTTAATGGCTGAGAGTGCT |
| | Reverse | CGGAAGGGGGACTTTGAACA |
| **Havcr2-5 (mouse)** | Forward | TGCACCCACCACCTGTAATG |
| | Reverse | CTCAGCTGTCATCCCTGGTC |
| **Havcr2-6 (mouse)** | Forward | GGAGAGTGACCTGACTACACC |
| | Reverse | GTCATGGCAAGAGGAGAGAGT |
| **Havcr2 (human)** | Forward | CTGCTGCTACTACTTACAAGGTC |

| | | |
|---|---|---|
| | Reverse | GCAGGGCAGATAGGCATTCT |
| **β-actin (mouse)** | Forward | GGCTGTATTCCCCTCCATCG |
| | Reverse | CCAGTTGGTAACAATGCCATGT |
| **β-actin (human)** | Forward | CCTTCCTGGGCATGGAGTCCT |
| | Reverse | GGAGCAATGATCTTGATCTT |
| **miR-4524a-5p (human)** | Forward | TTGACGATAGCAGCATGAACCT |
| | Reverse | TATCCTTCTTCACGACTCCTTCAC |
| **U6 (human)** | Forward | CAGCACATATACTAAAATTGGAACG |
| | Reverse | ACGAATTTGCGTGTCATCC |

## References


1. Shi W, Wang Y, Zhang C, et al. Isolation and purification of immune cells from the liver. Int Immunopharmacol 2020; **85**:106632.
2. Li PZ, Li JZ, Li M, et al. An efficient method to isolate and culture mouse Kupffer cells. Immunol Lett 2014; **158**:52-6.
