# Supplementary material for: Macrophage miR-4524a-5p/TBP promotes β-TrCP -TIM3 complex activation and TGFβ release and aggravates NAFLD-associated fibrosis
Source: Cell Death Dis. 2025 Apr 19;16(1):315. doi: 10.1038/s41419-025-07574-4 (PMC12008196; doi:10.1038/s41419-025-07574-4)

Figure 1F

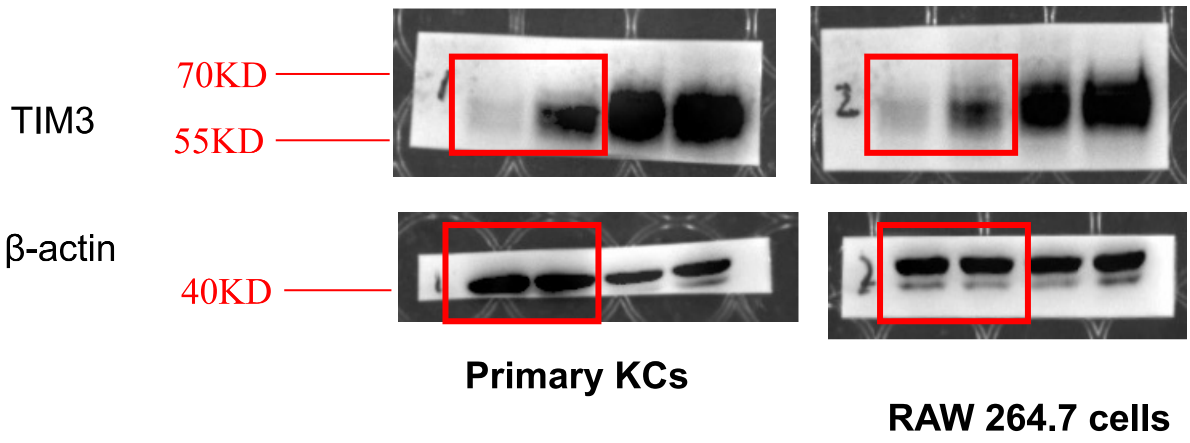

Figure 2A

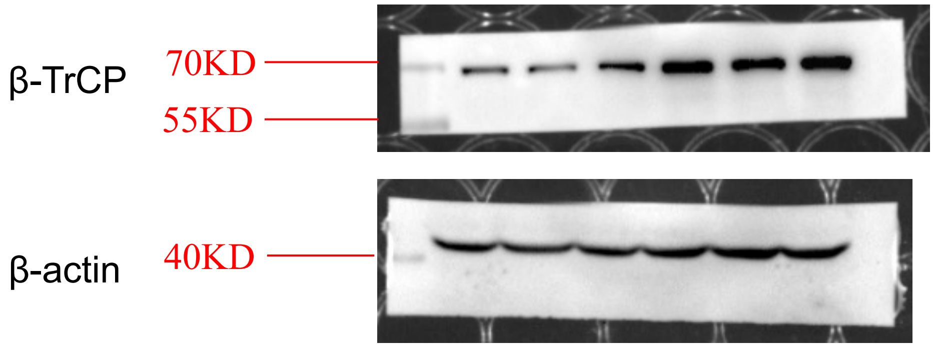

Figure 2B

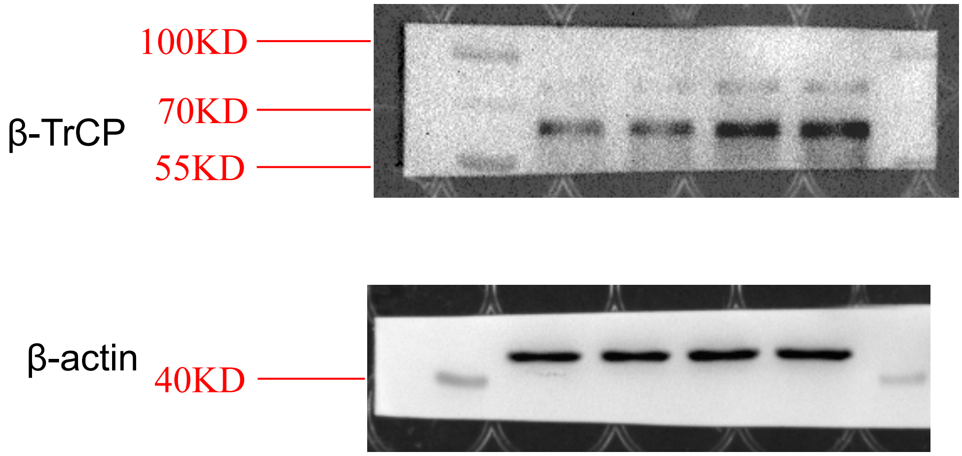

Figure 2C

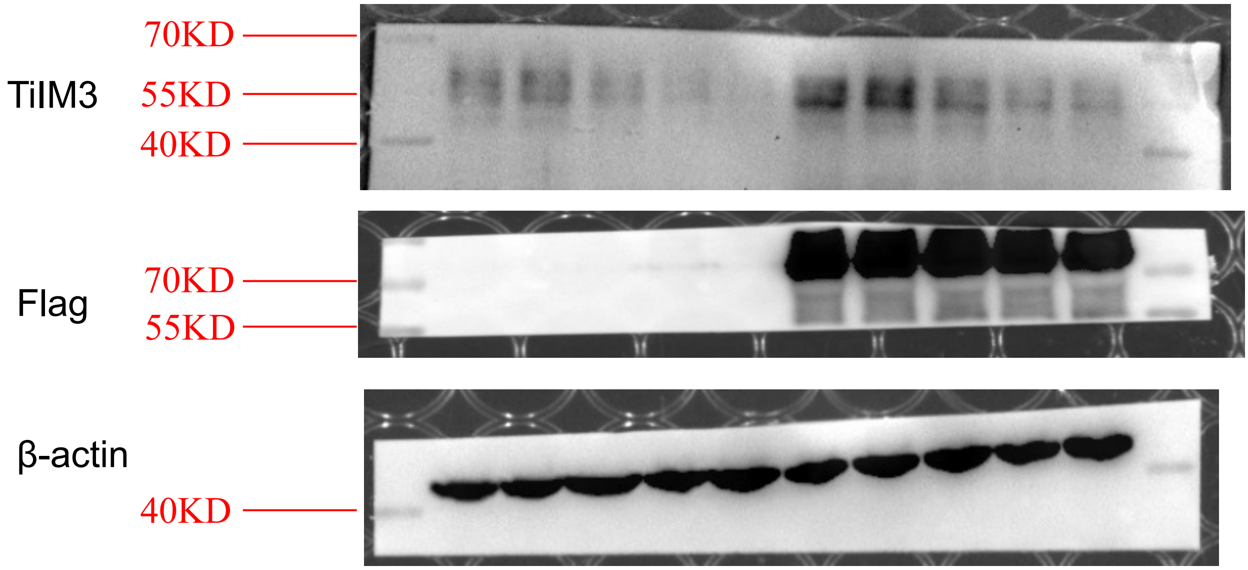

Figure 2D

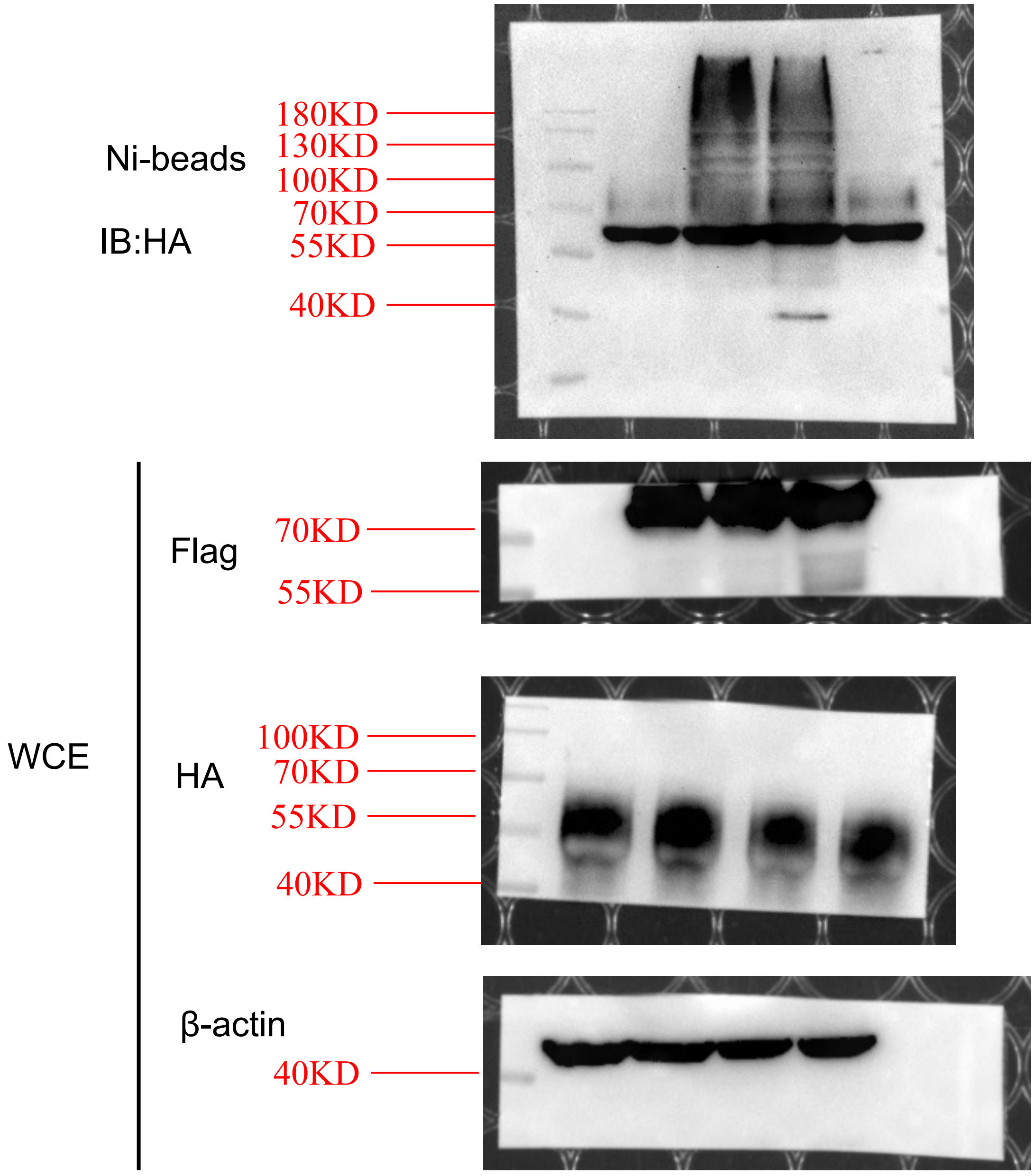

Figure 2E

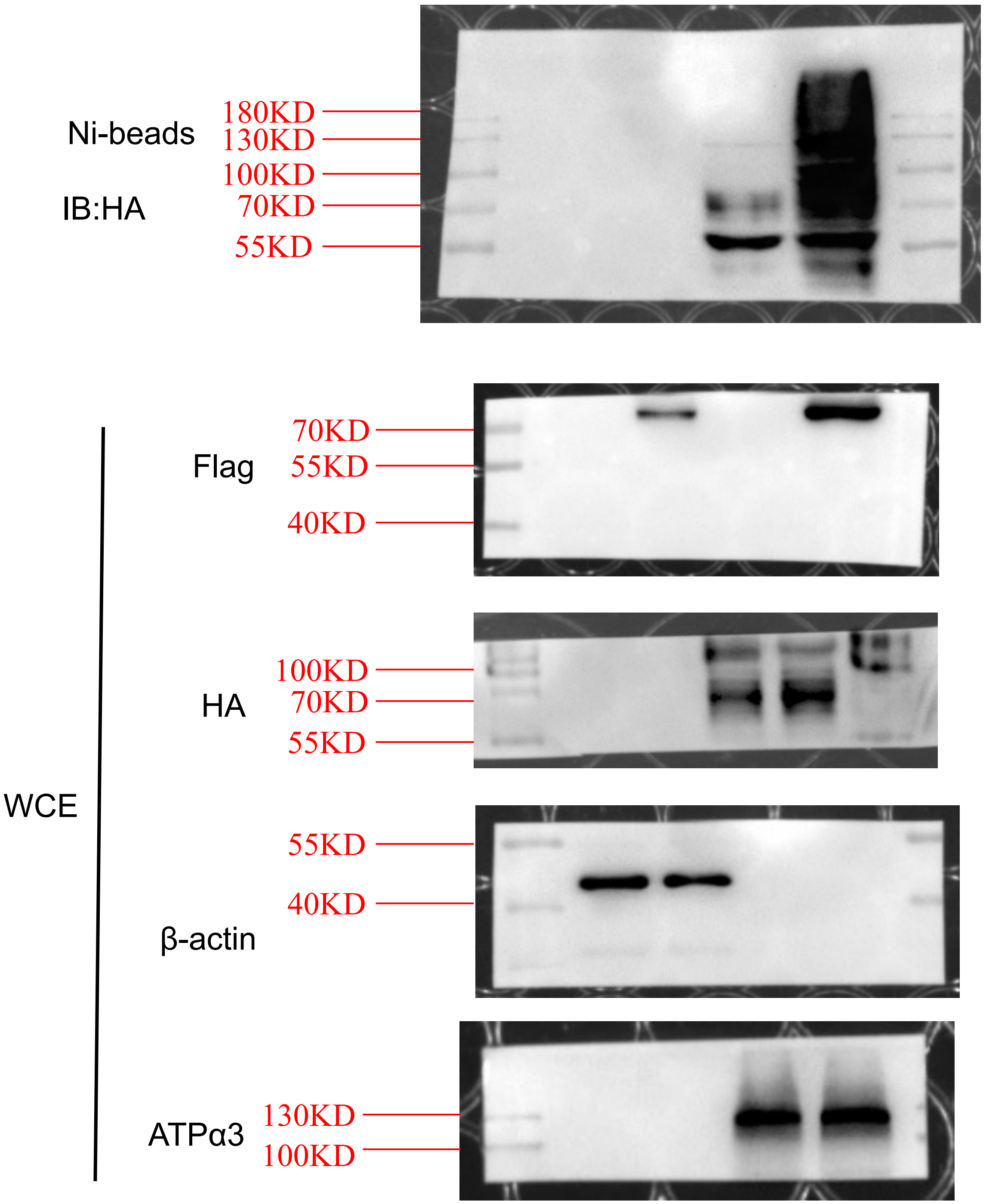

Ni-beads
IB:HA
180KD
130KD
100KD
70KD
55KD
WCE
Flag
70KD
55KD
40KD
HA
100KD
70KD
55KD
β-actin
55KD
40KD
ATPα3
130KD
100KD

Figure 2F

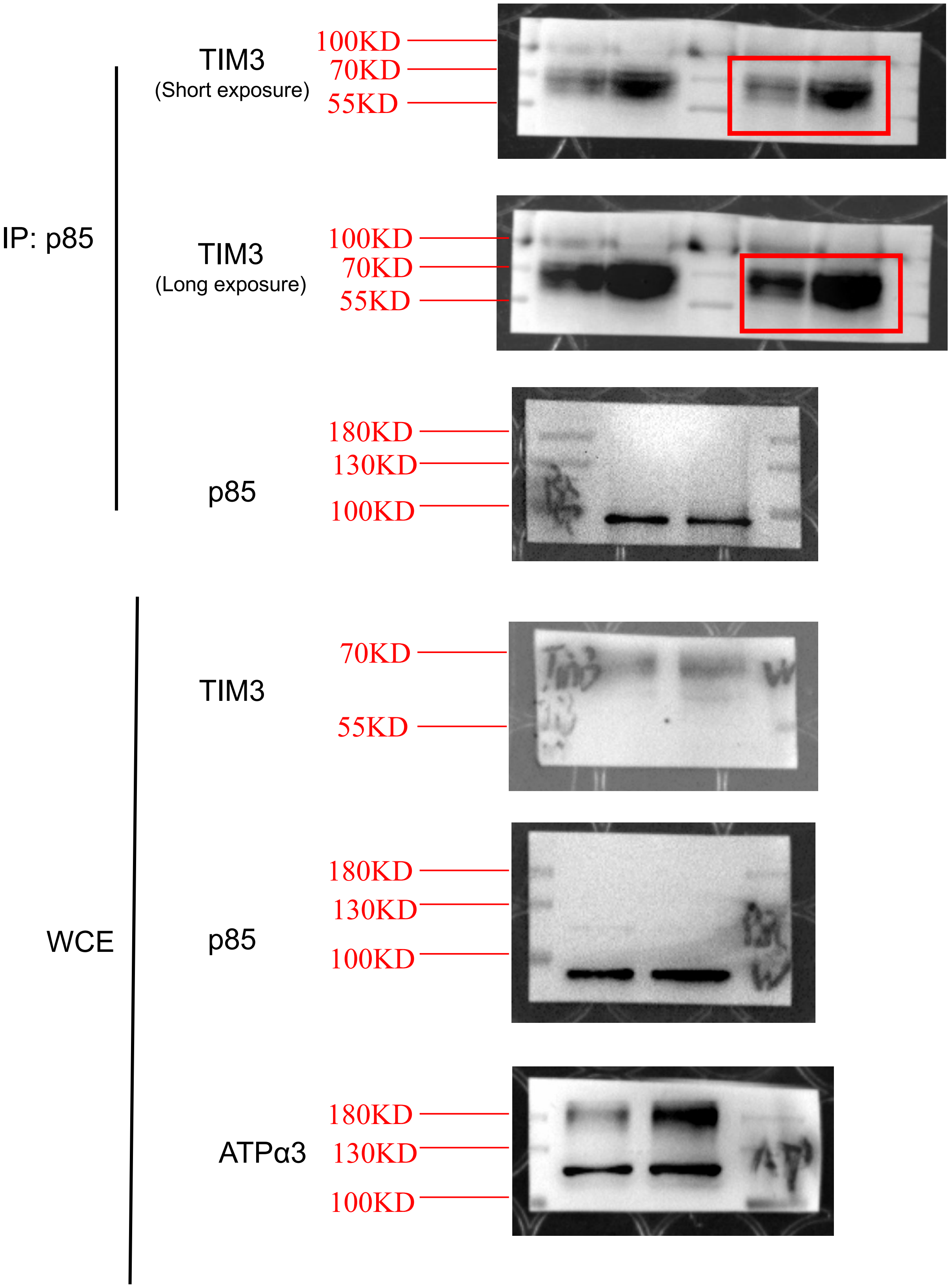
IP: p85
TIM3
(Short exposure)
100KD
70KD
55KD
TIM3
(Long exposure)
100KD
70KD
55KD
p85
180KD
130KD
100KD
WCE
TIM3
70KD
55KD
p85
180KD
130KD
100KD
ATPα3
180KD
130KD
100KD

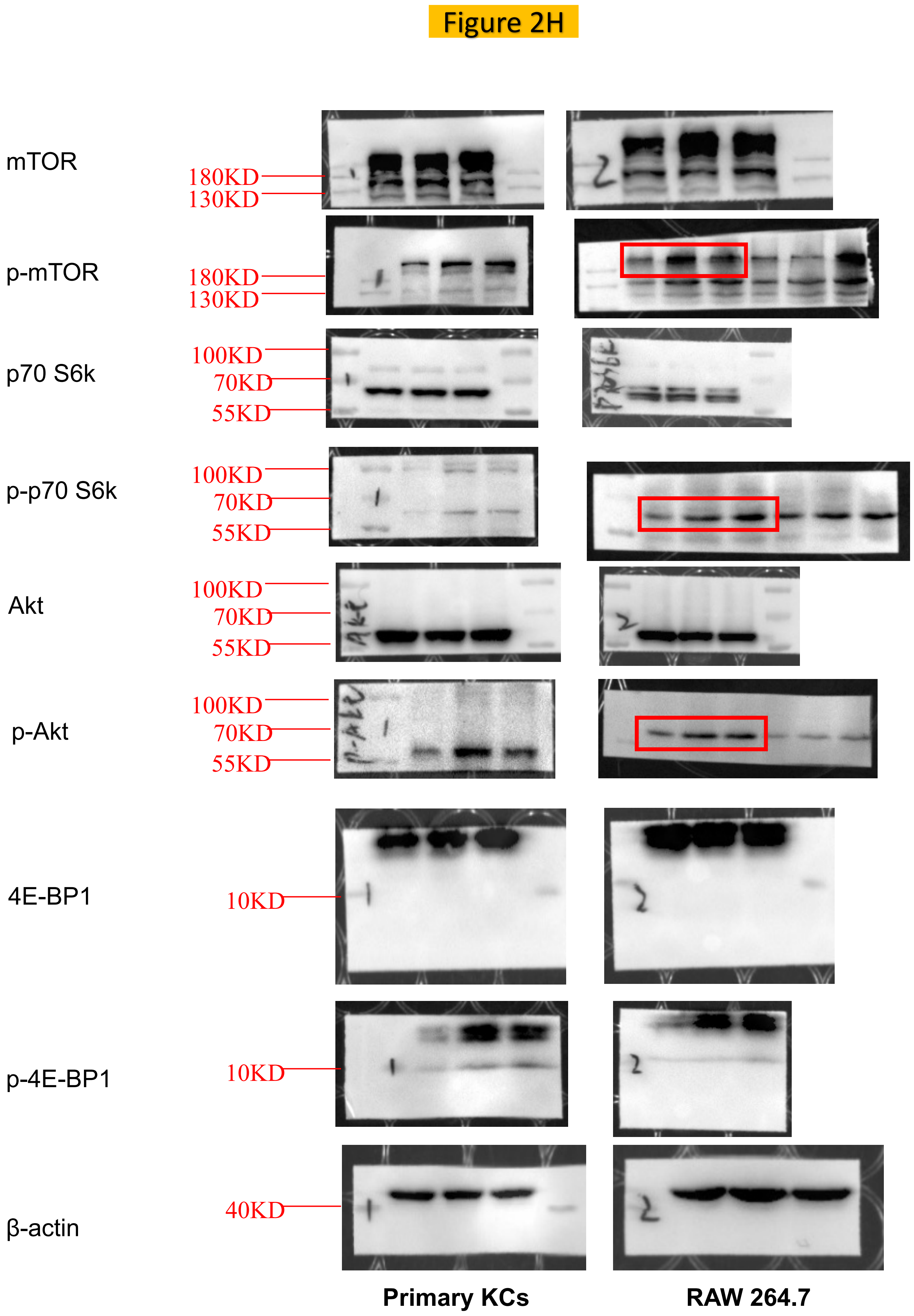
Figure 2H
mTOR
180KD
130KD
p-mTOR
180KD
130KD
p70 S6k
100KD
70KD
55KD
p-p70 S6k
100KD
70KD
55KD
Akt
100KD
70KD
55KD
p-Akt
100KD
70KD
55KD
4E-BP1
10KD
p-4E-BP1
10KD
β-actin
40KD
Primary KCs
RAW 264.7

Figure 3D

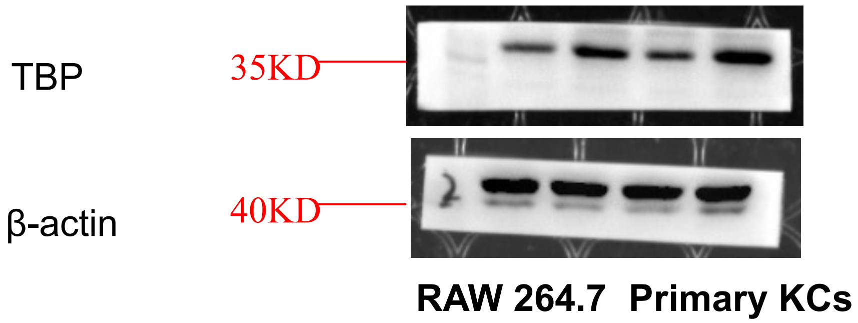

Figure 3H

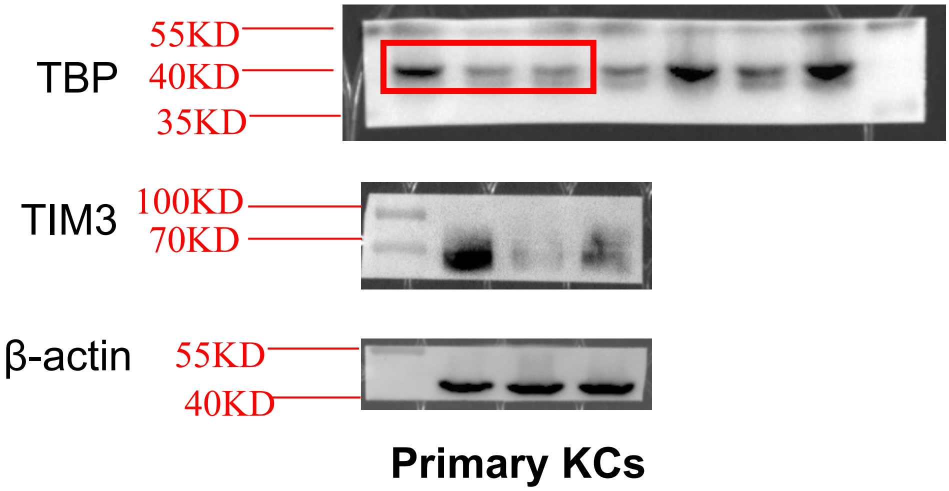

Figure 3I

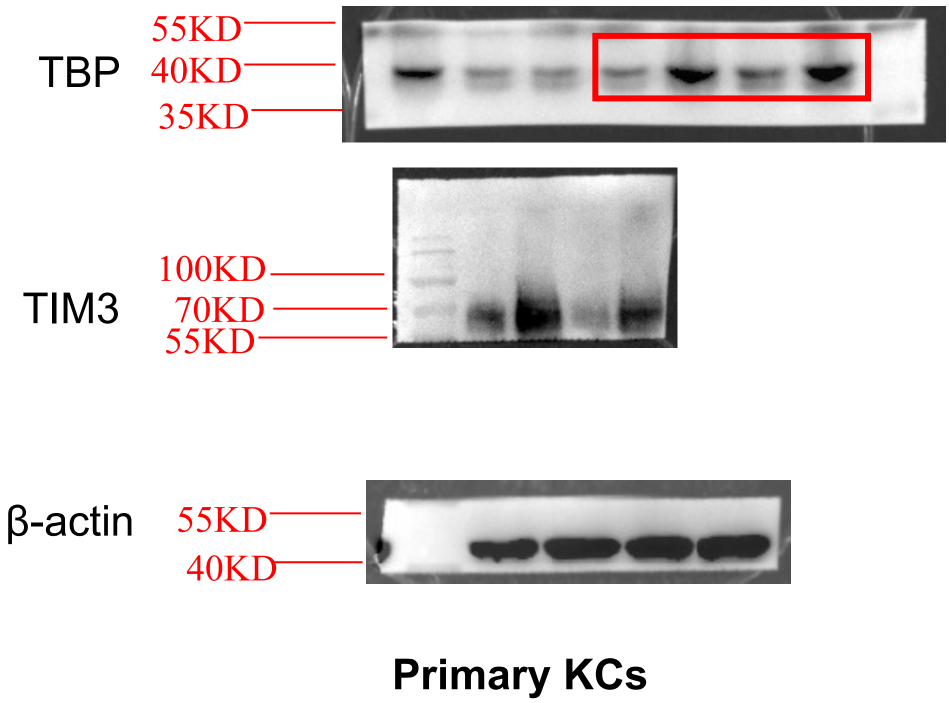

Figure 4E

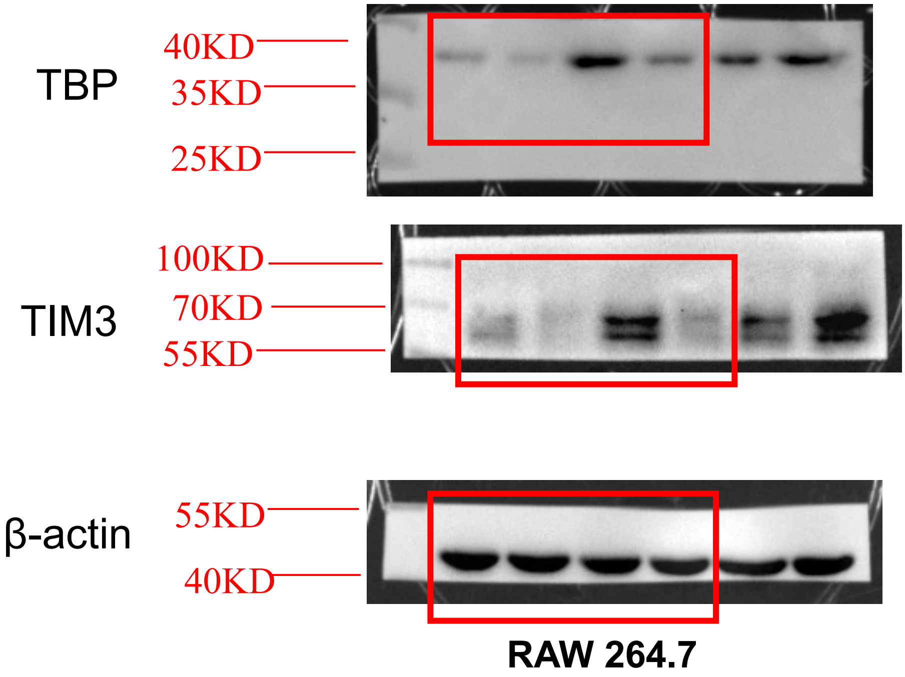

Figure 4G

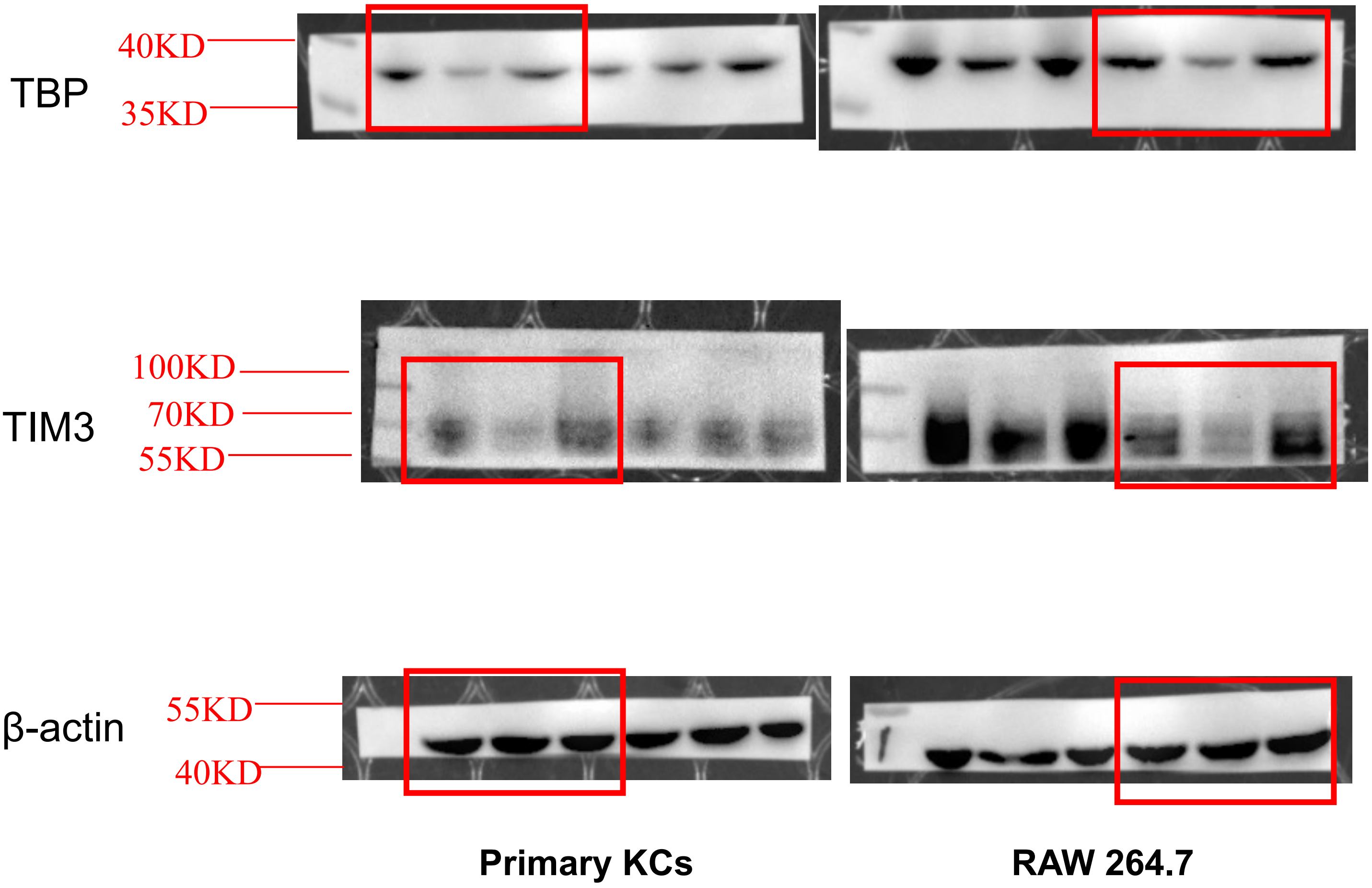

Figure 5C

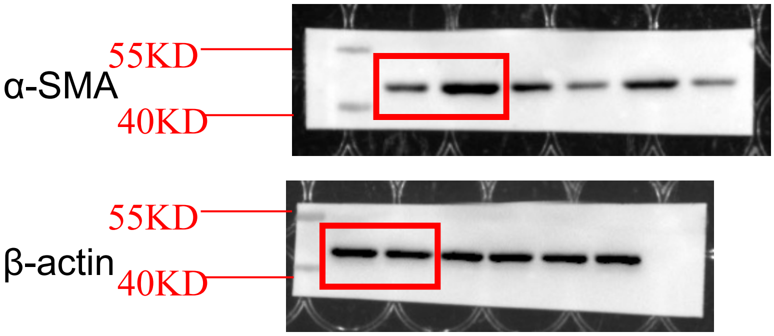

Figure 5E

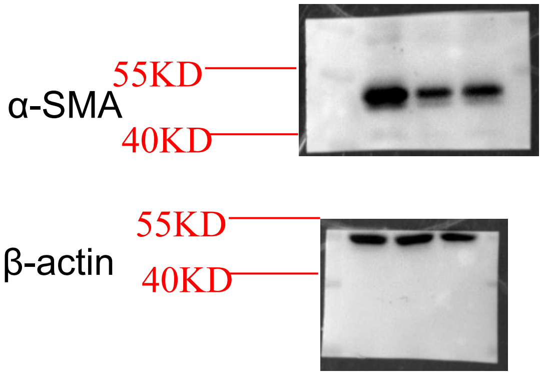

Figure 5H

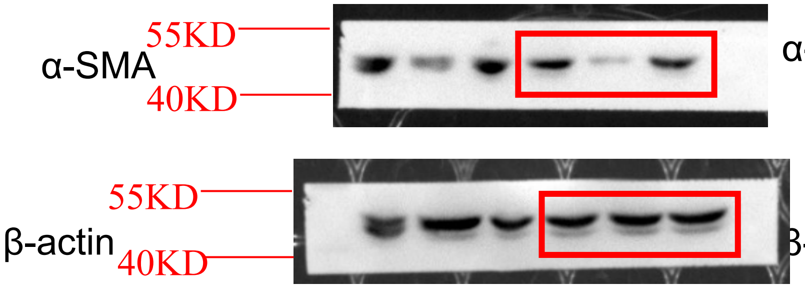

Figure 5J

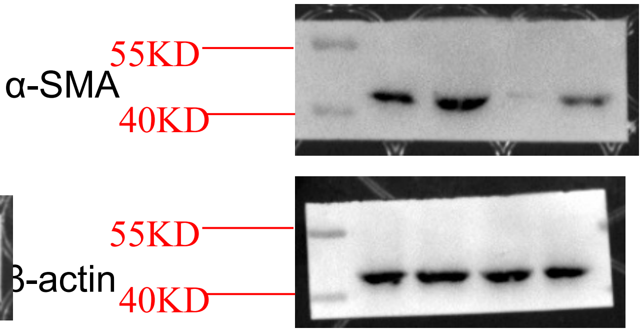

Figure 5M

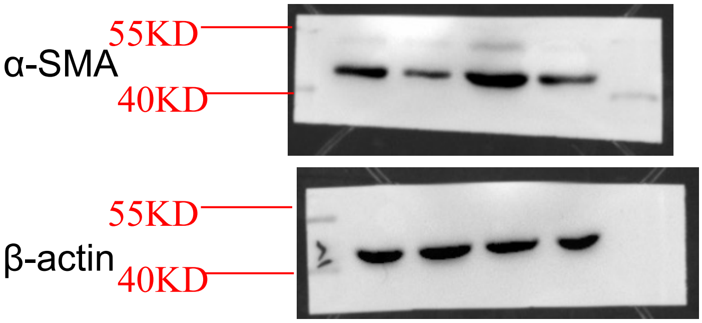

Figure 5O

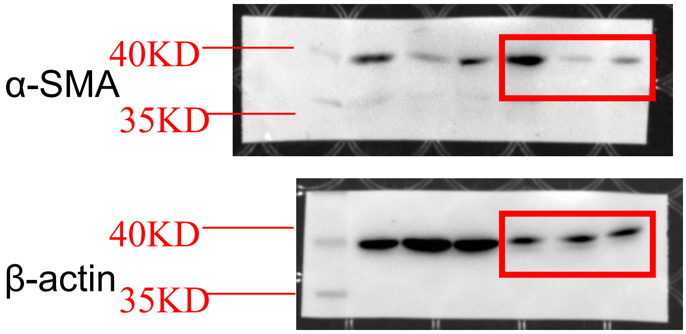

Figure S2E

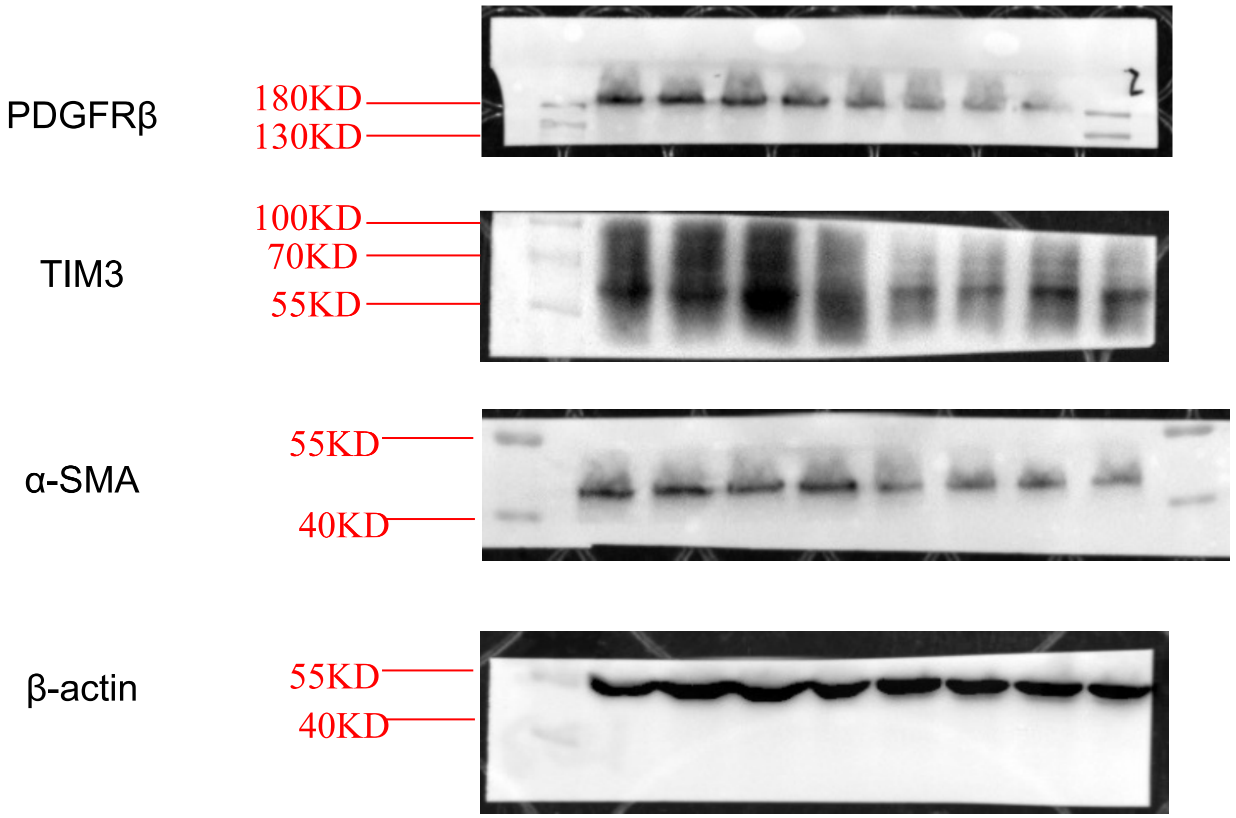

Figure S2L

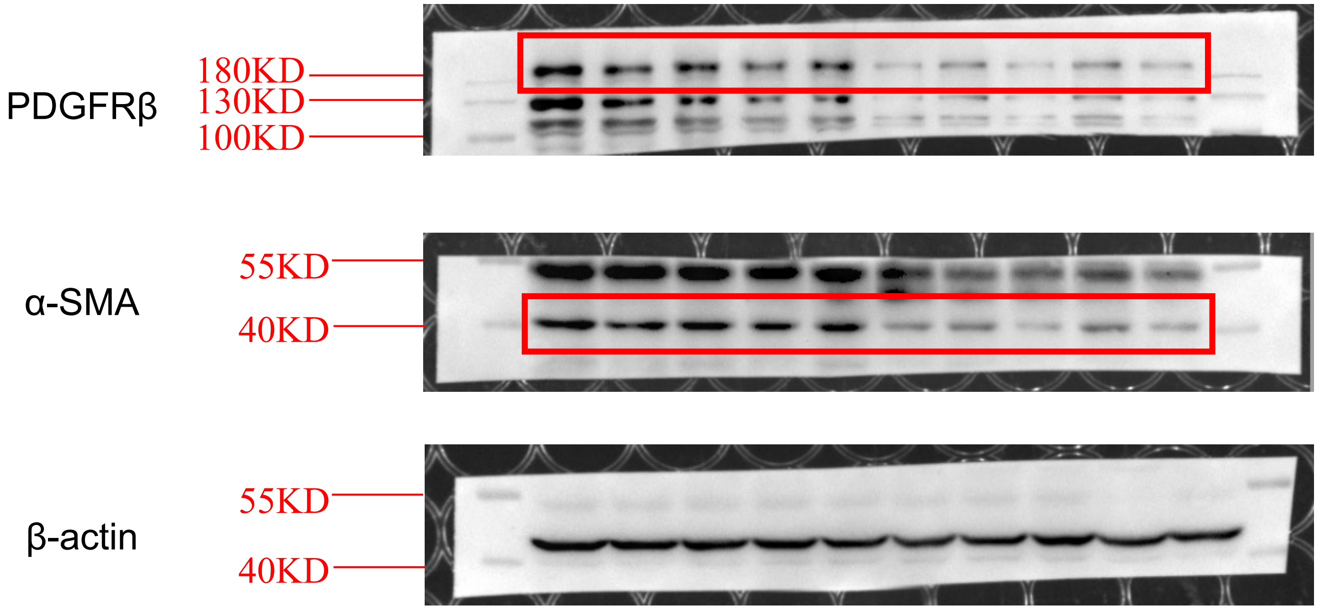

Figure S3B,C

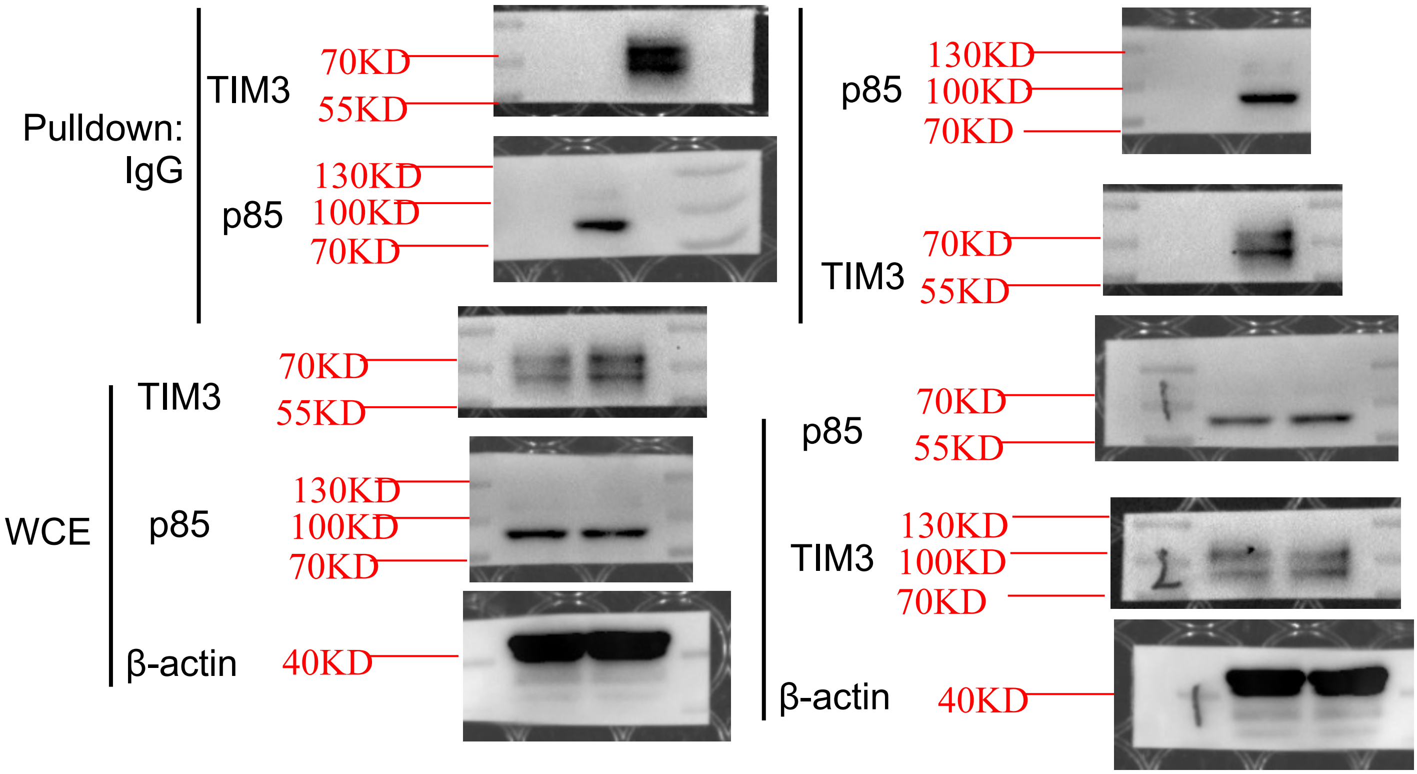

Figure S4C

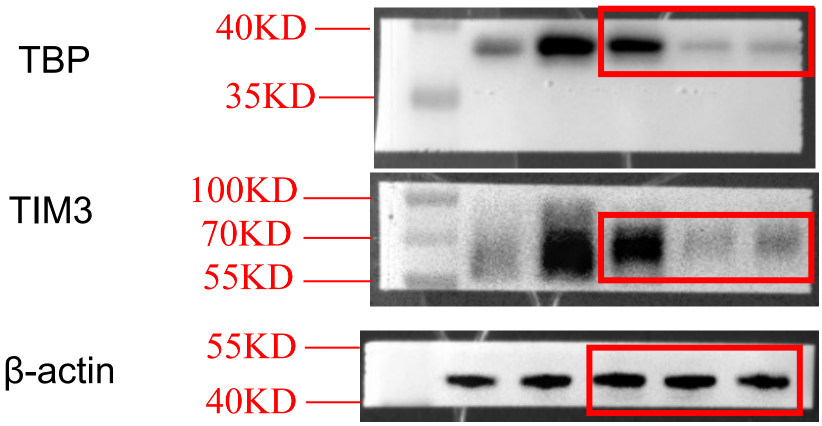

Figure S4E

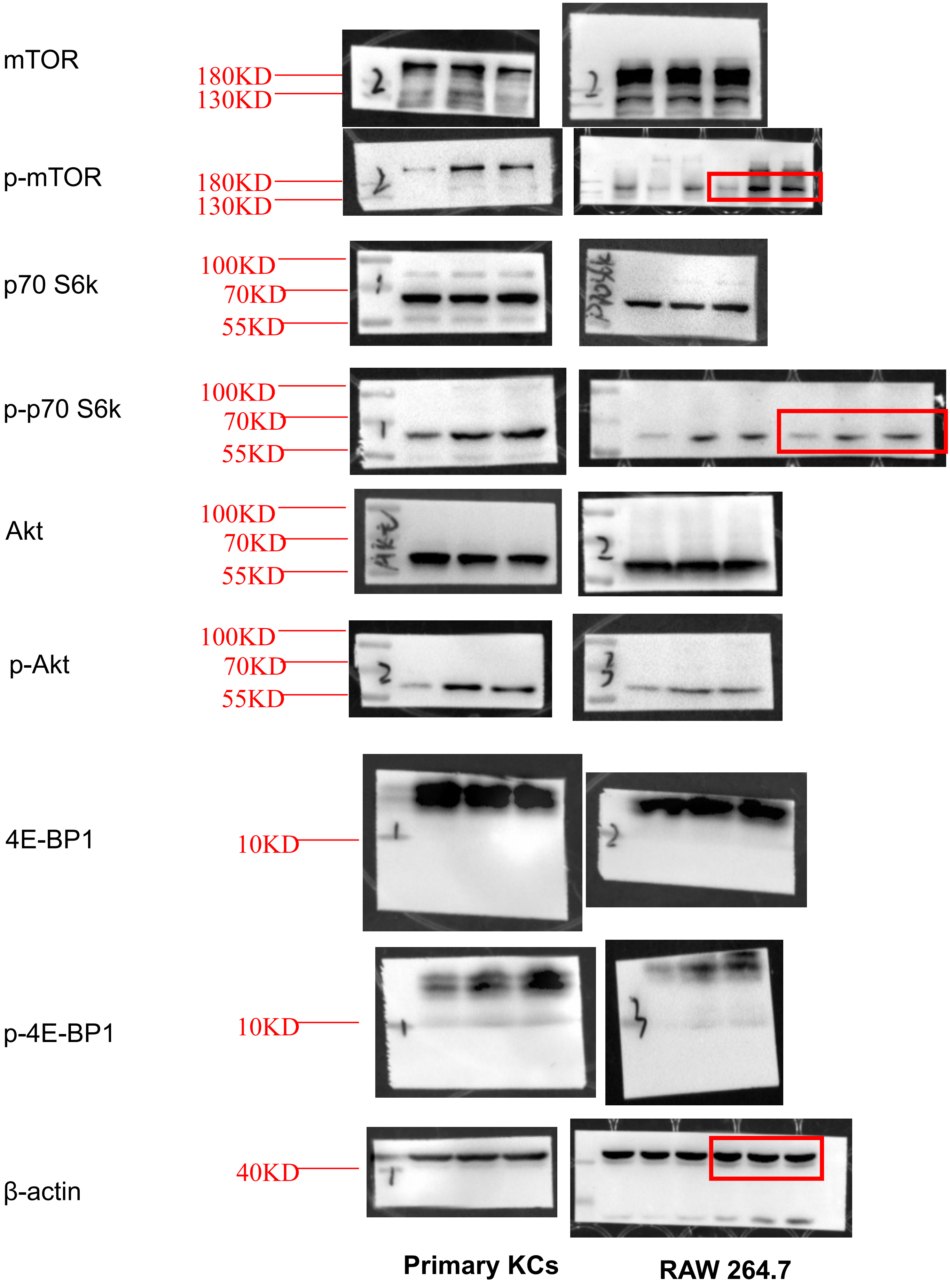
mTOR
180KD
130KD
p-mTOR
180KD
130KD
p70 S6k
100KD
70KD
55KD
p-p70 S6k
100KD
70KD
55KD
Akt
100KD
70KD
55KD
p-Akt
100KD
70KD
55KD
4E-BP1
10KD
p-4E-BP1
10KD
β-actin
40KD
Primary KCs
RAW 264.7

Figure S4H

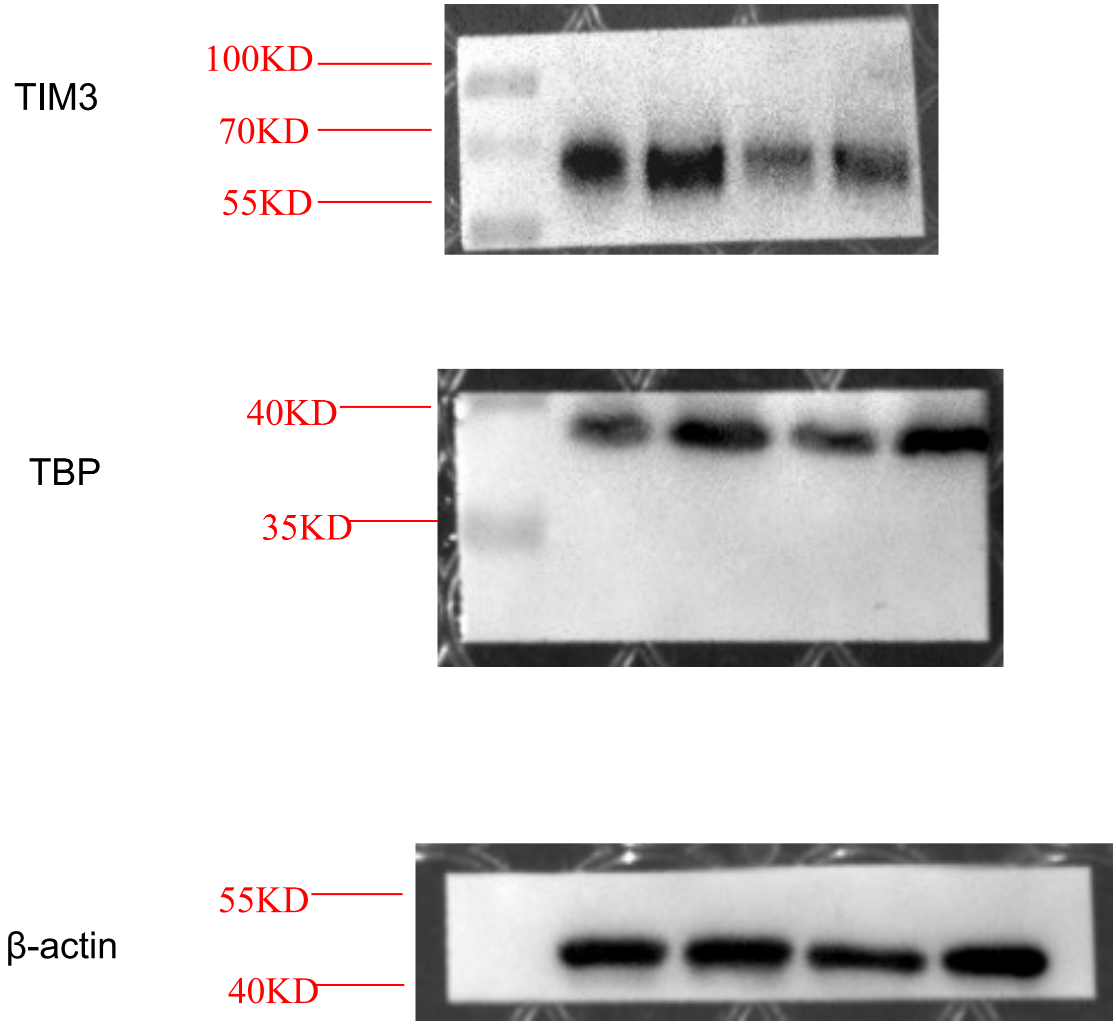

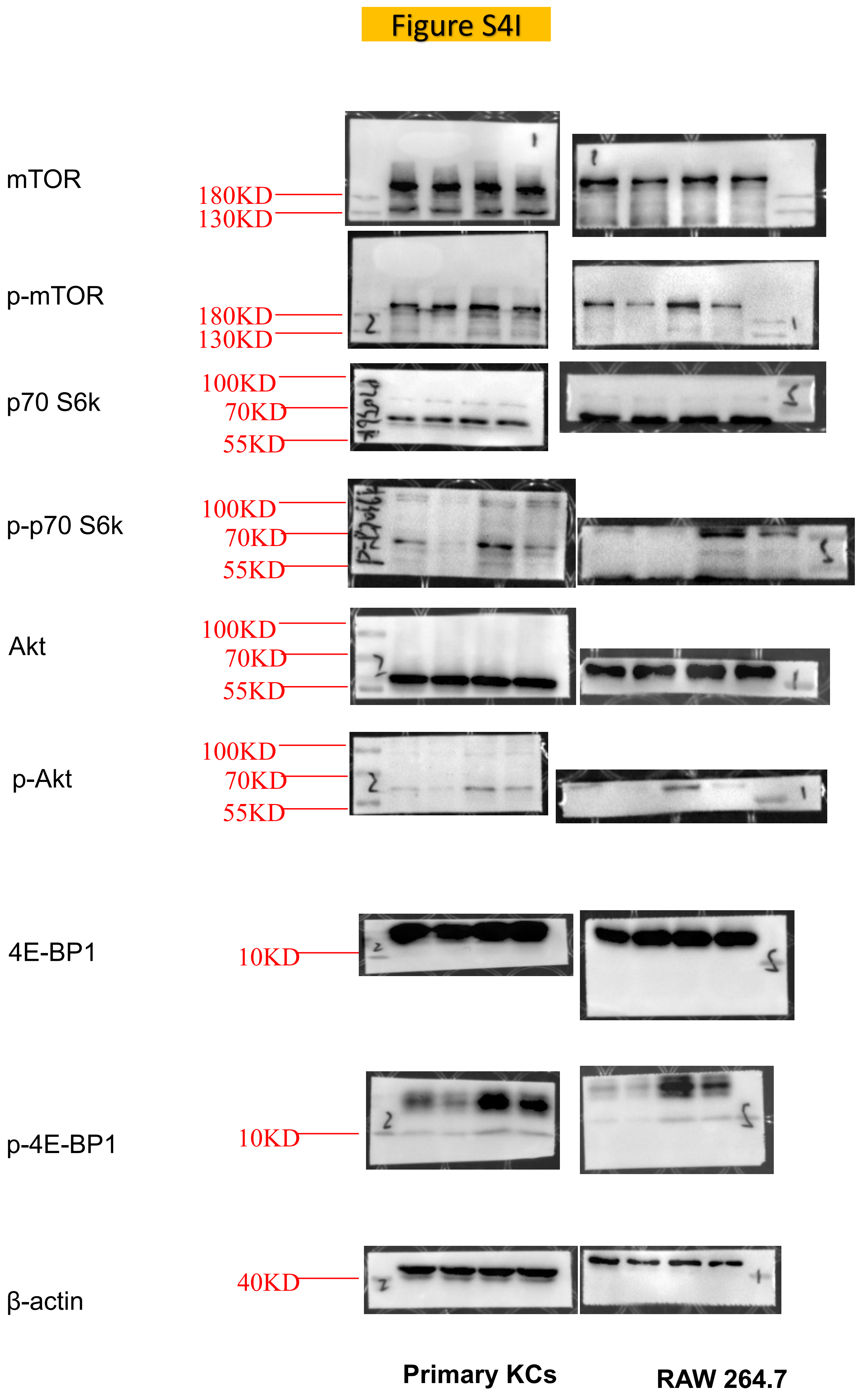
Figure S4I
mTOR
180KD
130KD
p-mTOR
180KD
130KD
p70 S6k
100KD
70KD
55KD
p-p70 S6k
100KD
70KD
55KD
Akt
100KD
70KD
55KD
p-Akt
100KD
70KD
55KD
4E-BP1
10KD
p-4E-BP1
10KD
β-actin
40KD
Primary KCs
RAW 264.7

Figure S6B

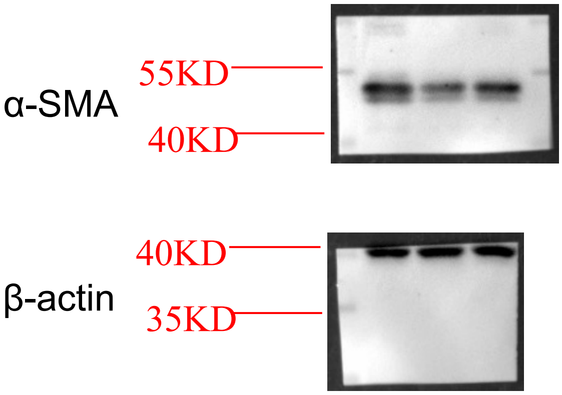

Figure S6D

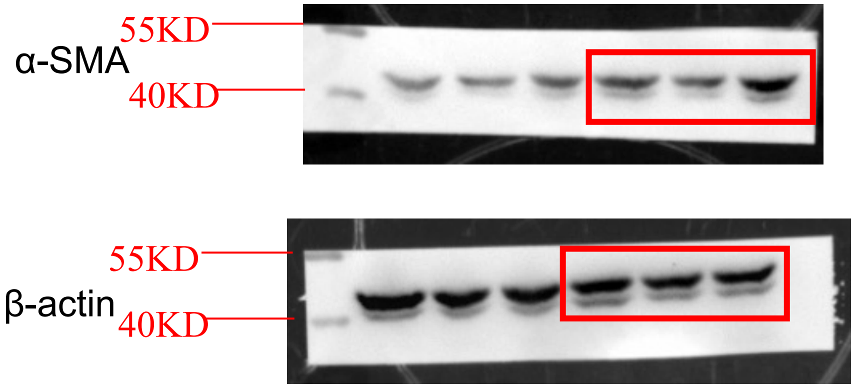

Figure S6F

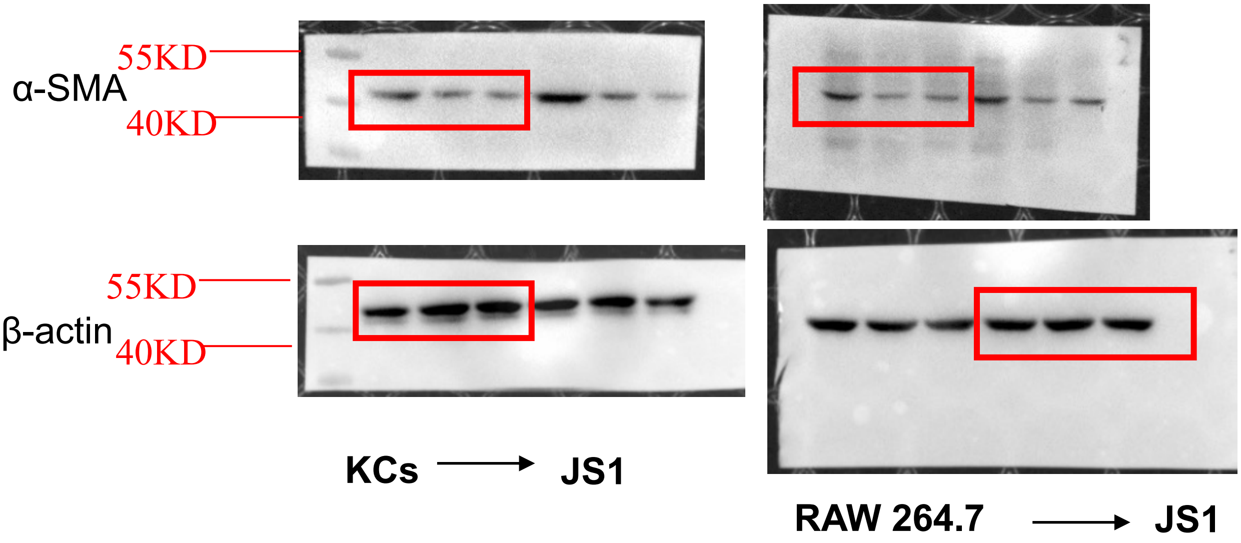

Figure S6H

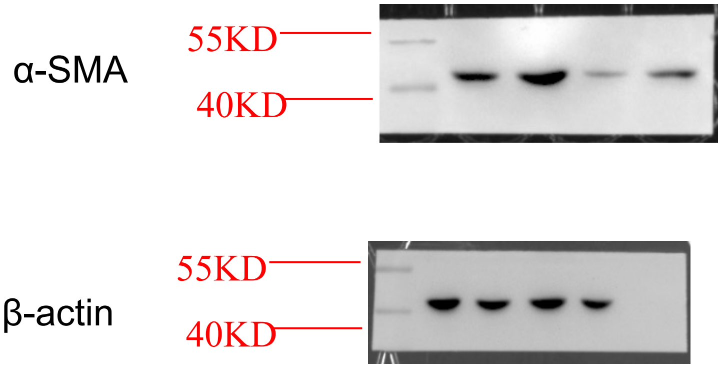

Figure S6K

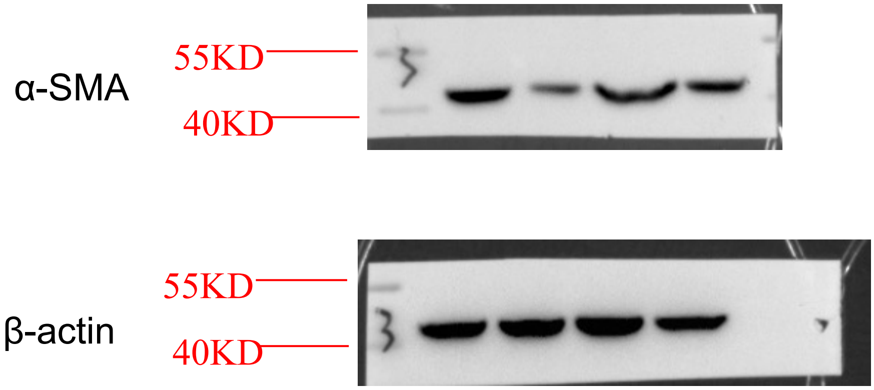

Figure S6M

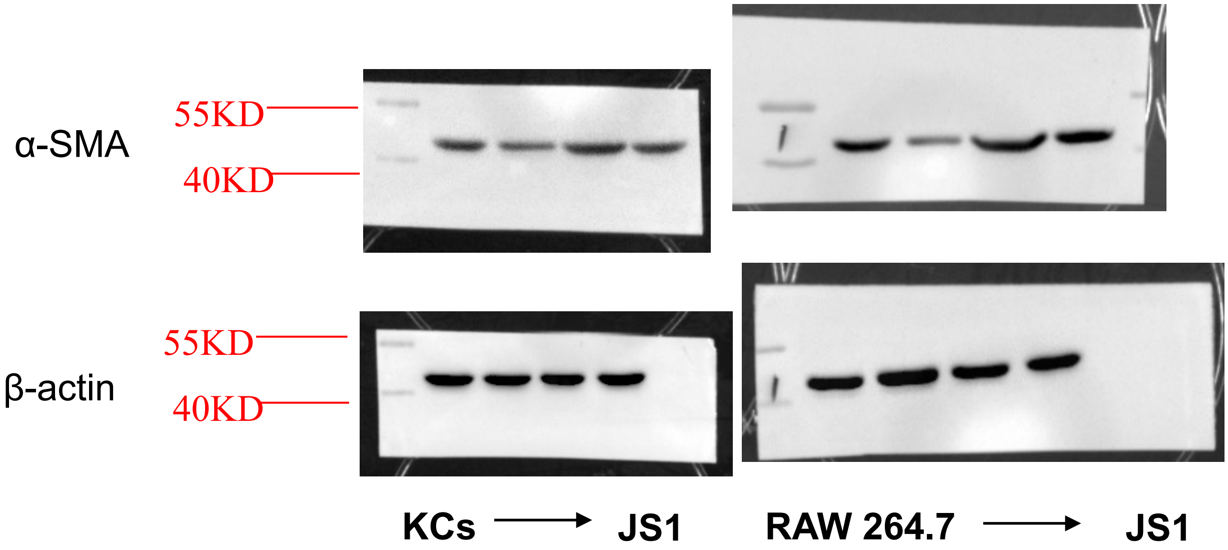

Figure S7A

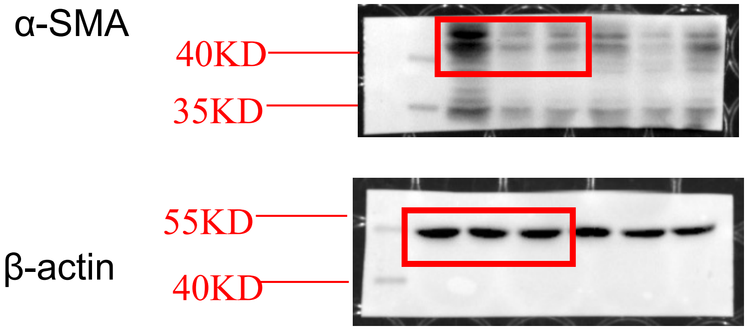

Figure S8E

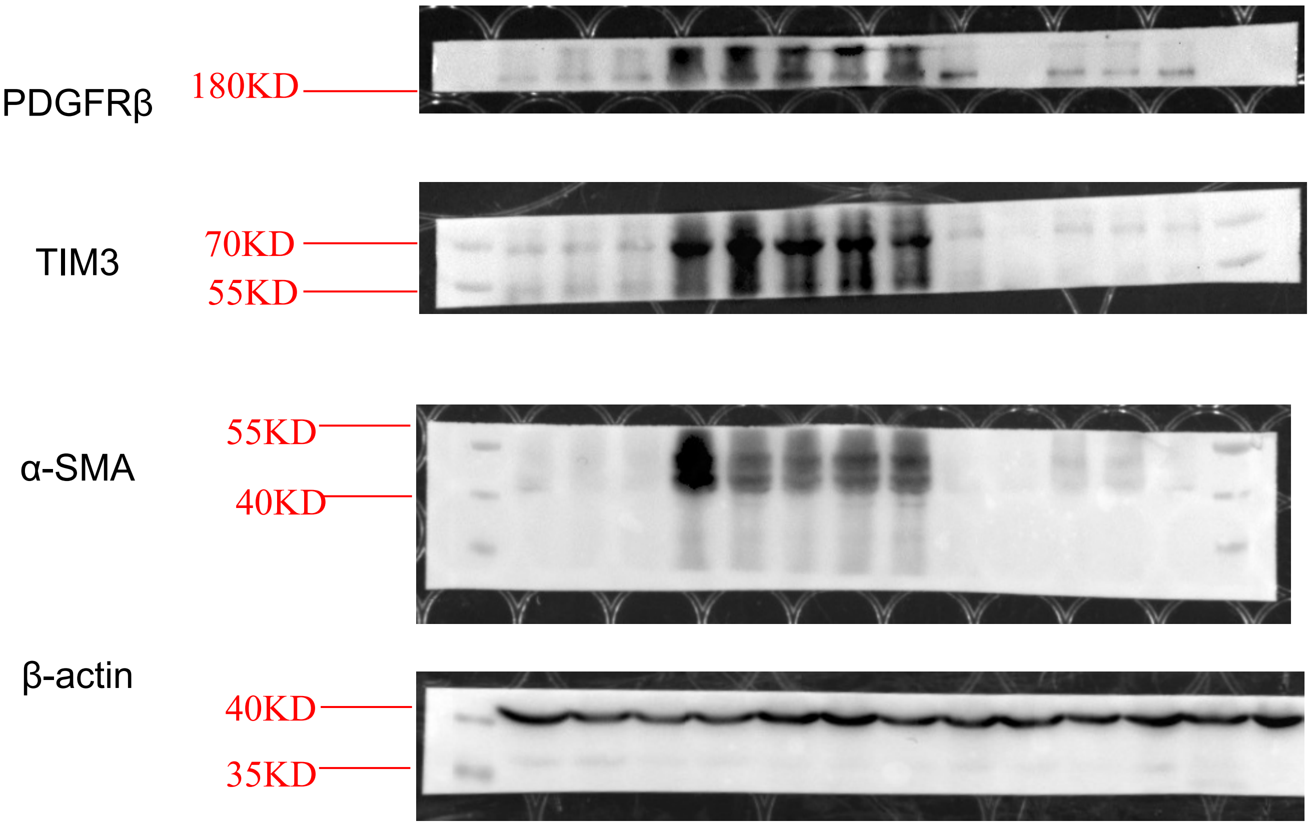

Supplement: Supplementary file 2 — Full and uncropped western blots [file 41419_2025_7574_MOESM2_ESM.pdf]
